# Influence of skeletal muscle and intermuscular fat on postoperative complications and long‐term survival in rectal cancer patients

**DOI:** 10.1002/jcsm.13424

**Published:** 2024-01-31

**Authors:** Tong Nie, Feihong Wu, Yixin Heng, Wentai Cai, Zhihao Liu, Le Qin, Yinghao Cao, Chuansheng Zheng

**Affiliations:** ^1^ Department of Radiology, Union Hospital, Tongji Medical College Huazhong University of Science and Technology Wuhan China; ^2^ Hubei Province Key Laboratory of Molecular Imaging Wuhan China; ^3^ Department of General Surgery The First Affiliated Hospital of Shihezi University Shihezi China; ^4^ The First Clinical School, Union Hospital, Tongji Medical College Huazhong University of Science and Technology Wuhan China; ^5^ China Medical University Shenyang China; ^6^ Department of Gastrointestinal Surgery, Union Hospital, Tongji Medical College Huazhong University of Science and Technology Wuhan China; ^7^ Cancer Center, Union Hospital, Tongji Medical College Huazhong University of Science and Technology Wuhan China; ^8^ Department of Digestive Surgical Oncology, Cancer Center, Union Hospital, Tongji Medical College Huazhong University of Science and Technology Wuhan China

**Keywords:** Disease‐free survival, Intermuscular fat, Overall survival, Postoperative complications, Rectal cancer, Skeletal muscle density, Skeletal muscle index

## Abstract

**Background:**

The body composition of patients with rectal cancer potentially affects postoperative outcomes. This study explored the correlations between skeletal muscle and adipose tissue quantified by computed tomography (CT) with postoperative complications and long‐term prognosis in patients with rectal cancer after surgical resection.

**Methods:**

This retrospective cohort study included patients with rectal cancer who underwent surgical resection at the Wuhan Union Hospital between 2014 and 2018. CT images within 3 months prior to the surgery were used to quantify the indices of skeletal muscle and adipose tissue at the levels of the third lumbar vertebra (L3) and umbilicus. Optimal cut‐off values for each index were defined separately for males and females. Associations between body composition and postoperative complications, overall survival (OS), and disease‐free survival (DFS) were evaluated using logistic and Cox proportional hazards models.

**Results:**

We included 415 patients (240 males and 175 females; mean age: 57.8 ± 10.5 years). At the L3 level, a high skeletal muscle density (SMD; hazard ratio [HR]: 0.357, 95% confidence interval [CI]: 0.191–0.665, *P* = 0.001; HR: 0.571, 95% CI: 0.329–0.993, *P* = 0.047) and a high skeletal muscle index (SMI; HR: 0.435, 95% CI 0.254–0.747, *P* = 0.003; HR: 0.568, 95% CI: 0.359–0.897, *P* = 0.015) were independent prognostic factors for better OS and DFS. At the umbilical level, a large intermuscular fat area (IMFA; HR: 1.904, 95% CI: 1.068–3.395, *P* = 0.029; HR: 2.064, 95% CI: 1.299–3.280, *P* = 0.002) was an independent predictive factor for worse OS and DFS, and a high SMI (HR: 0.261, 95% CI: 0.132–0.517, *P* < 0.001; HR: 0.595, 95% CI: 0.387–0.913, *P* = 0.018) was an independent prognostic factor for better OS and DFS. The models combining body composition and clinical indicators had good predictive abilities for OS. The receiver operating characteristic areas under the curve were 0.848 and 0.860 at the L3 and umbilical levels, respectively (both *P* < 0.05).

**Conclusions:**

No correlations existed between CT‐quantified body composition parameters and postoperative complications. However, a high SMD and high SMI were significantly associated with longer OS and DFS at the L3 level, whereas a large IMFA and low SMI were associated with worse OS and DFS at the umbilical level. Combining CT‐quantified body composition and clinical indicators could help physicians predict the prognosis of patients with rectal cancer after surgery.

## Introduction

Colorectal cancer (CRC) is the third most common cancer worldwide, accounting for approximately 10% of all cancers, and is the second leading cause of cancer‐related deaths.^1^ Rectal cancer accounts for 37% of all CRC cases among individuals aged <50 years.^2^ Surgery is the most common treatment for rectal cancer, and in recent years, the 5‐year survival rate for rectal cancer has improved owing to advancements in treatment techniques and perioperative care.^3^ However, population growth and aging projections until 2035 foresee an increase in the number of rectal cancer deaths in all countries by 71.5%.^4^

Previous studies have suggested that body composition correlates with postoperative complications and long‐term prognosis in patients with CRC.^5^ Body composition is the proportion and distribution of tissues in the body, including bones, muscles, and fat.^6^, ^7^ In the past, weight and body mass index (BMI, weight [kg]/height [m^2^]) were the most commonly used indicators for measuring overall body composition. However, their use as prognostic indicators was limited because weight and BMI cannot measure fat distribution or distinguish between the masses of muscle and fat.^8^ Increasing evidence suggests that computed tomography (CT) scanning provides a more accurate reflection of skeletal muscle and adipose tissue. However, studies on the correlation between adipose tissue, postoperative complications, and long‐term prognosis for patients with CRC have produced inconsistent results.^9^, ^10^ In addition, several recent studies have reported significant associations between skeletal muscle depletion and density and unfavourable outcomes after surgical resection for CRC.^11^, ^12^, ^13^, ^14^ Skeletal muscle depletion is the disproportionate loss of skeletal muscle, related to an imbalance between protein synthesis and breakdown, resulting in sarcopenia.^15^ The main reason for decreased skeletal muscle density is myosteatosis, which manifests as the accumulation of inter‐ and intramuscular fat in the muscles.^16^ Therefore, measuring intermuscular adipose tissue can indirectly reflect skeletal muscle density. The evidence regarding the effects of body composition on the complications and prognosis in patients with CRC is inconsistent, and few studies have focused explicitly on rectal cancer. In addition, most of the research has been based on Western populations^17^, ^18^; few studies on Asian populations exist.

This study explored the impact of CT‐quantified body composition on the postoperative complications and long‐term prognosis of rectal cancer, aiming to establish prediction models that combine body composition and clinical indicators to improve postoperative prognoses for patients with rectal cancer.

## Methods

### Patient selection

We included patients with confirmed rectal cancer who underwent surgical resection at Wuhan Union Hospital between 2014 and 2018. The inclusion criteria were as follows: (1) pathologically confirmed rectal cancer; (2) age ≥18 years; (3) an abdominal CT scan performed within 3 months before surgery. The exclusion criteria were as follows: (1) incomplete clinical data; (2) multiple metastases or recurrent rectal cancer; (3) loss to follow‐up or inability to obtain follow‐up outcomes; and (4) poor CT image quality due to severe artefacts or extreme emaciation, which makes it difficult to distinguish between fat and muscle (Figure S1). Informed consent was obtained from all patients, and the study was approved by the Medical Ethics Committee of Wuhan Union Hospital (No. 2018‐S377).

### Clinical variables

Clinical data of patients were obtained from the electronic medical record system, including sex, age, BMI, family history of cancer, history of previous abdominal surgery, neoadjuvant treatment, co‐morbidities (including cardiovascular disease, cerebrovascular disease, chronic obstructive pulmonary disease [COPD], and diabetes), tumour size, history of preoperative obstruction, postoperative radiotherapy, postoperative chemotherapy, lymphovascular invasion (LVI), nerve invasion, histological grade, tumour node metastasis (TNM) stage, and serum tumour markers (carcinoembryonic antigen [CEA], carbohydrate antigen [CA19‐9, CA125, and CA72‐4]). Perioperative data included the surgery type, blood transfusion, primary anastomosis, and colostomy. Short‐term outcomes included postoperative complications, including obstruction, anastomotic fistula, local infection, thrombosis, cardio‐cerebrovascular disease, and length of stay (LOS).

### Study endpoints

The primary endpoint was overall survival (OS), which is defined as the time from surgery to death or the end of follow‐up. The secondary endpoint was disease‐free survival (DFS), which is defined as the time from the day of surgery to tumour recurrence, metastasis, or the end of follow‐up.

### Patient follow‐up

A follow‐up protocol was established following the Chinese Society of Clinical Oncology Guidelines for the Diagnosis and Treatment of CRC. Starting on postoperative day one, patients were followed up either by phone or in person at the hospital. Follow‐up visits were scheduled every 3 months for the first 3 years after surgery, and every 6 months for the fourth and fifth years. Patients who failed to attend their appointment within 1 year of their last visit were considered lost to follow‐up. In addition, chest and abdominal‐pelvic CT scans were performed every 6 or 12 months for 5 years, depending on the patient's pathological stage, to determine whether recurrence or metastasis had occurred. The collected follow‐up information included the adjuvant therapy status (radiotherapy or chemotherapy administration), tumour recurrence, time of recurrence (if applicable), patient survival time, and time of death.

### Body composition analysis

**Figure 1:**
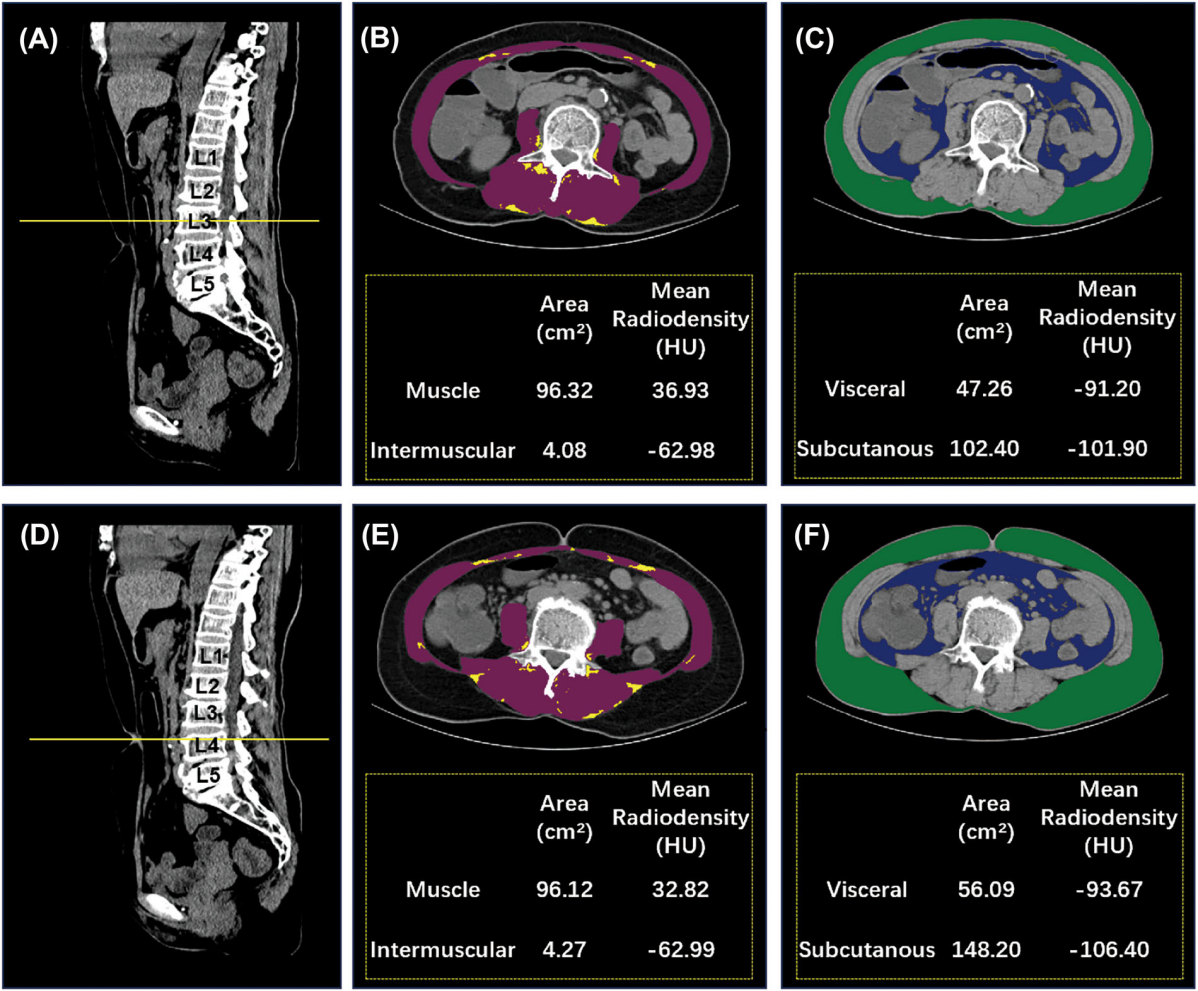
Body morphometric evaluations of abdominal fat and muscle areas at the L3 level and umbilical level. Axial slices of a male patient at the L3 and umbilical levels. (A) Sagittal reformation of a preoperative CT scan at the L3 level. (B, C) skeletal muscle area (in red) is 96.32 cm^2^; intermuscular fat area (in yellow) is 4.08 cm^2^; visceral fat area (in blue) is 47.26 cm^2^; subcutaneous fat area (in green) is 102.40 cm^2^. (D) Sagittal reformation on preoperative CT scan at the umbilical level. (E, F) skeletal muscle area (in red) is 96.12 cm^2^; intermuscular fat area (in yellow) is 4.27 cm^2^; visceral fat area (in blue) is 56.09 cm^2^; subcutaneous fat area (in green) is 148.20 cm^2^. CT, computed tomography; L3, lumbar 3 vertebra.

A single CT image at the third lumbar vertebra (L3) level was selected to quantify skeletal muscle and adipose tissue (Figure 1A–C), in view of previous studies, which closely correlates with the total body composition volume.^19^ In addition, we chose a single CT image at the umbilical level as a supplement (Figure 1D–F). According to the standard Hounsfield unit (HU) range, the visceral fat area (VFA), subcutaneous fat area (SFA), intermuscular fat area (IMFA), skeletal muscle area (SMA), and skeletal muscle density (SMD) were measured using SliceOmatic version 5.0 (TomoVision, Magog, Quebec, Canada). Based on previous studies,^20^ we set thresholds of −150 HU to −50 HU for visceral fat, −190 HU to −30 HU for subcutaneous and intermuscular fat, and −29 HU to 150 HU for skeletal muscle. The SMD was automatically generated as the mean radiation attenuation of the muscle region of interest. Skeletal muscle mass was calculated as the skeletal muscle index (SMI) from the total muscle cross‐sectional area divided by height squared.

### Optimal cut‐off points for body composition

X‐tile software^21^ (version 3.6.1; Yale University School of Medicine; New Haven, CT, USA) was used to determine the optimal VFA, SFA, IMFA, SMA, SMD, and SMI cut‐off values. Differences in skeletal muscle and adipose tissue distribution between male and female patients exist.^22^ Therefore, the patients were divided into high and low body composition groups based on sex‐specific cut‐off points. The cut‐off values for tumour size, CEA, CA19‐9, CA125, CA72‐4, and LOS were also calculated.

### Statistical analyses

Statistical analyses were performed using SPSS (version 25.0; Armonk, NY, USA), GraphPad Prism 9 (GraphPad Inc., San Diego, CA, USA), and R software (version 4.2.2; R Core Team, Vienna, Austria). Count data were represented as percentages (*N* %), and continuous data were expressed as mean ± standard deviations (SD). Comparative analyses of baseline data between groups were performed using the *χ* ^2^ test or Fisher's exact test for qualitative variables and the Mann–Whitney *U* test for continuous variables. Kaplan–Meier survival curves were plotted and compared using the log‐rank tests. Logistic regression analyses were conducted to evaluate the associations between body composition and postoperative complications. Cox proportional hazards models were used to assess the correlations between body composition and long‐term survival. Variables with *P*‐values of <0.05 in the univariate analysis were entered into the multivariate analysis. The results are reported as hazard ratios (HRs) and corresponding 95% confidence intervals (CIs). Predictive models for OS and DFS were established; Model 1 included the TNM stage, Model 2 included the TNM stage and body composition parameters (VFA, IMFA, SMA, SFA, SMD, and SMI), and Model 3 included the TNM stage and independent prognostic factors (body composition and clinical indicators). Receiver operating characteristic (ROC) curves and the area under the ROC curve (AUC) were used to assess the predictive performance of the prognostic factors in different models. Calibration curves were used to assess the concordance between predicted and observed risks. Statistical significance was established set at *P* < 0.05.

## Results

### Population characteristics

This study included 415 patients. The mean age was 57.8 ± 10.5 years, and 57.8% were man. Figure S2 (L3 level) and Figure S3 (umbilical level) present the optimal body composition cut‐off values. At the L3 level, IMFA values of >3.23 cm^2^ and >2.29 cm^2^ were considered high for male and female patients, respectively. SMI values of ≤39.23 cm^2^/m^2^ and ≤36.70 cm^2^/m^2^ and SMD values of ≤29.32 HU and ≤22.80 HU were considered low for male and female patients, respectively. The general clinical characteristics of the patients with low and high body composition indices were inconsistent between the two anatomical locations.

### Clinical characteristics based on skeletal muscle density and skeletal muscle index

Table 1 presents the general clinical characteristics of the high and low SMD groups, which were between the two anatomical locations. At the L3 level, 10.4% (mean: 25.56 ± 4.44 HU) and 10.3% (mean: 19.72 ± 3.37 HU) of male and female patients, respectively, were in the low SMD group. More patients were older (69.8% vs. 23.9%, *P* < 0.001) and had a history of abdominal surgery (27.9%), any co‐morbidities (48.8%), and a high CA125 level (30.2%) in the low SMD group than in the high SMD group (all *P* < 0.05).

**Table 1:** Patient characteristics based on SMD

	*N* (%)						
	Overall	L3 SMD		P	Umbilical SMD		*P*
Characteristics	(*N* = 415)	Low (*n* = 43)	High (*n* = 372)		Low (*n* = 83)	High (*n* = 332)	
Sex							
Male	240 (57.8)	25 (58.1)	215 (57.8)	0.966	63 (75.9)	177 (53.3)	**<0.001**
Female	175 (42.2)	18 (41.9)	157 (42.2)		20 (24.1)	155 (46.7)	
Age (years)							
<65	296 (71.3)	13 (30.2)	283 (76.1)	**<0.001**	40 (48.2)	256 (77.1)	**<0.001**
≥65	119 (28.7)	30 (69.8)	89 (23.9)		43 (51.8)	76 (22.9)	
BMI (kg/m^2^)							
<25	341 (82.2)	37 (86.0)	304 (81.7)	0.483	65 (78.3)	276 (83.1)	0.305
≥25	74 (17.8)	6 (14.0)	68 (18.3)		18 (21.7)	56 (16.9)	
Obstruction before surgery							
Absent	394 (94.9)	38 (88.4)	356 (95.7)	0.088	81 (97.6)	313 (94.3)	0.341
Present	21 (5.1)	5 (11.6)	16 (4.3)		2 (2.4)	19 (5.7)	
Family history							
No	392 (94.5)	41 (95.3)	351 (94.4)	1.000	75 (90.4)	317 (95.5)	0.120
Yes	23 (5.5)	2 (4.7)	21 (5.6)		8 (9.6)	15 (4.5)	
Radiotherapy							
No	386 (93.0)	40 (93.0)	346 (93.0)	1.000	77 (92.8)	309 (93.1)	0.923
Yes	29 (7.0)	3 (7.0)	26 (7.0)		6 (7.2)	23 (6.9)	
Chemotherapy							
No	168 (40.5)	20 (46.5)	148 (39.8)	0.395	40 (48.2)	128 (38.6)	0.110
Yes	247 (59.5)	23 (53.5)	224 (60.2)		43 (51.8)	204 (61.4)	
Neoadjuvant treatment							
No	383 (92.3)	37 (86.0)	346 (93.0)	0.187	74 (89.2)	309 (93.1)	0.232
Yes	32 (7.7)	6 (14.0)	26 (7.0)		9 (10.8)	23 (6.9)	
Tumour size (cm)							
≤2.6	96 (23.1)	11 (25.6)	85 (22.8)	0.688	19 (22.9)	77 (23.2)	0.954
>2.6	319 (76.9)	32 (74.4)	287 (77.2)		64 (77.1)	255 (76.8)	
LVI							
Absent	339 (81.7)	31 (72.1)	308 (82.8)	0.086	67 (80.7)	272 (81.9)	0.800
Present	76 (18.3)	12 (27.9)	64 (17.2)		16 (19.3)	60 (18.1)	
Nerve invasion							
Absent	329 (79.3)	30 (69.8)	299 (80.4)	0.104	57 (68.7)	272 (81.9)	**0.008**
Present	86 (20.7)	13 (30.2)	73 (19.6)		26 (31.3)	60 (18.1)	
Histological grade							
Poor	51 (12.3)	5 (11.6)	46 (12.4)	0.501	9 (10.8)	42 (12.7)	0.212
Moderate	302 (72.8)	34 (79.1)	268 (72.0)		68 (81.9)	234 (70.5)	
Well	62 (14.9)	4 (9.3)	58 (15.6)		6 (7.2)	56 (16.9)	
Stage							
I	109 (26.3)	6 (14.0)	103 (27.7)	**0.048**	19 (22.9)	90 (27.1)	0.707
II	109 (26.3)	11 (25.6)	98 (26.3)		25 (30.1)	84 (25.3)	
III	157 (37.8)	21 (48.8)	136 (36.6)		30 (36.1)	127 (38.3)	
IV	40 (9.6)	5 (11.6)	35 (9.4)		9 (10.8)	31 (9.3)	
Tumour							
T1	38 (9.2)	3 (7.0)	35 (9.4)	0.065	6 (7.2)	32 (9.6)	0.208
T2	94 (22.7)	6 (14.0)	88 (23.7)		15 (18.1)	79 (23.8)	
T3	241 (58.1)	27 (62.8)	214 (57.5)		53 (63.9)	188 (56.6)	
T4	42 (10.1)	7 (16.3)	35 (9.4)		9 (10.8)	33 (9.9)	
Nodes							
N0	232 (55.9)	20 (46.5)	212 (57.0)	0.104	47 (56.6)	185 (55.7)	0.828
N1	109 (26.3)	11 (25.6)	98 (26.3)		18 (21.7)	91 (27.4)	
N2	74 (17.8)	12 (27.9)	62 (16.7)		18 (21.7)	56 (16.9)	
Metastasis							
M0	377 (90.8)	39 (90.7)	338 (90.9)	0.972	74 (89.2)	303 (91.3)	0.552
M1	38 (9.2)	4 (9.3)	34 (9.1)		9 (10.8)	29 (8.7)	
Previous abdominal surgery							
No	362 (87.2)	31 (72.1)	331 (89.0)	**0.002**	70 (84.3)	292 (88.0)	0.378
Yes	53 (12.8)	12 (27.9)	41 (11.0)		13 (15.7)	40 (12.0)	
Any co‐morbidities							
No	302 (72.8)	22 (51.2)	280 (75.3)	**0.001**	53 (63.9)	249 (75.0)	**0.041**
Yes	113 (27.2)	21 (48.8)	92 (24.7)		30 (36.1)	83 (25.0)	
CEA (ng/mL)							
≤11.6	344 (82.9)	31 (72.1)	313 (84.1)	**0.047**	61 (73.5)	283 (85.2)	**0.011**
>11.6	71 (17.1)	12 (27.9)	59 (15.9)		22 (26.5)	49 (14.8)	
CA19–9 (kU/L)							
≤53.2	373 (89.9)	35 (81.4)	338 (90.9)	0.093	72 (86.7)	301 (90.7)	0.290
>53.2	42 (10.1)	8 (18.6)	34 (9.1)		11 (13.3)	31 (9.3)	
CA125 (U/mL)							
≤15.9	345 (83.1)	30 (69.8)	315 (84.7)	**0.013**	67 (80.7)	278 (83.7)	0.512
>15.9	70 (16.9)	13 (30.2)	57 (15.3)		16 (19.3)	54 (16.3)	
CA72–4 (U/mL)							
≤9.4	367 (88.4)	37 (86.0)	330 (88.7)	0.791	72 (86.7)	295 (88.9)	0.591
>9.4	48 (11.6)	6 (14.0)	42 (11.3)		11 (13.3)	37 (11.1)	

Bold was used to highlight values that were statistically significant (*P* < 0.05).

Any co‐morbidities, including cardiovascular disease, cerebrovascular disease, COPD, and diabetes; BMI, body mass index (weight [kg]/height [m^2^]); CA19‐9; CA125; CA72‐4, carbohydrate antigen; CEA, carcino‐embryonic antigen; COPD, chronic obstructive pulmonary disease; LVI, lymphovascular invasion; SMD, skeletal muscle density.

At the umbilical level, 26.2% and 11.4% of male and female patients, respectively, were in the low SMD group. More patients were older and male in the low SMD group than in the high SMD group (51.8% vs. 22.9%; 75.9% vs. 53.3%, both *P* < 0.001). More patients had nerve invasion (31.3%), any co‐morbidities (36.1%), and a higher CEA level (26.5%) in the low SMD group than in the high SMD group (all *P* < 0.05).

Table S1 presents the general clinical characteristics of the high and low SMI groups. At the L3 level, more patients were older and female in the low SMI group than in the high SMI group (43.5% vs. 24.5%; 71.7% vs. 33.7%, both *P* < 0.001). Most patients had a BMI of <25 kg/m^2^ (93.5%), a history of abdominal surgery (23.9%) and a high CA125 level (26.1%) in the low SMI group than in the high SMI group (all *P* < 0.05). At the umbilical level, more patients were older and male in the low SMI group than in the high SMI group (45.3% vs. 19.5%; 83.8% vs. 43.4%, both *P* < 0.001). Most patients had a BMI of <25 kg/m^2^ (91.2%) in the low SMI group than in the high SMI group (*P* < 0.001).

### Clinical characteristics based on intermuscular fat area

Table 2 presents the general clinical characteristics of the high and low IMFA groups. At the L3 level, 77.5% and 89.1% of male and female patients, respectively, were in the high IMFA group. Most patients were older and female in the high IMFA group than in the low IMFA group (32.5% vs. 11.0%, *P* < 0.001; 45.6% vs. 26.0%, *P* = 0.002). More patients did not receive chemotherapy (43.9%) and had TNM stages I and II (28.1% and 26.3%) disease in the high IMFA group than in the low group.

**Table 2:** Patient characteristics based on IMFA

Characteristics	*N* (%)						
	Overall (*N* = 415)	L3 IMFA		*P*	Umbilical IMFA		*P*
		Low (*n* = 73)	High (*n* = 342)		Low (*n* = 227)	High (*n* = 188)	
Sex							
Male	240 (57.8)	54 (74.0)	186 (54.4)	**0.002**	108 (47.6)	132 (70.2)	**<0.001**
Female	175 (42.2)	19 (26.0)	156 (45.6)		119 (52.4)	56 (29.8)	
Age (years)							
<65	296 (71.3)	65 (89.0)	231 (67.5)	**<0.001**	182 (80.2)	114 (60.6)	**<0.001**
≥65	119 (28.7)	8 (11.0)	111 (32.5)		45 (19.8)	74 (39.4)	
BMI (kg/m^2^)							
<25	341 (82.2)	64 (87.7)	277 (81.0)	0.176	195 (85.9)	146 (77.7)	**0.029**
≥25	74 (17.8)	9 (12.3)	65 (19.0)		32 (14.1)	42 (22.3)	
Obstruction before surgery							
Absent	394 (94.9)	70 (95.9)	324 (94.7)	0.909	215 (94.7)	179 (95.2)	0.817
Present	21 (5.1)	3 (4.1)	18 (5.3)		12 (5.3)	9 (4.8)	
Family history							
No	392 (94.5)	2 (2.7)	21 (6.1)	0.249	9 (4.0)	14 (7.4)	0.123
Yes	23 (5.5)	71 (97.3)	321 (93.9)		218 (96.0)	174 (92.6)	
Radiotherapy							
No	386 (93.0)	68 (93.2)	318 (93.0)	0.959	211 (93.0)	175 (93.1)	0.958
Yes	29 (7.0)	5 (6.8)	24 (7.0)		16 (7.0)	13 (6.9)	
Chemotherapy							
No	168 (40.5)	18 (24.7)	150 (43.9)	**0.002**	85 (37.4)	83 (44.1)	0.166
Yes	247 (59.5)	55 (75.3)	192 (56.1)		142 (62.6)	105 (55.9)	
Neoadjuvant treatment							
No	383 (92.3)	67 (91.8)	316 (92.4)	0.858	212 (93.4)	171 (91.0)	0.355
Yes	32 (7.7)	6 (8.2)	26 (7.6)		15 (6.6)	17 (9.0)	
Tumour size (cm)							
≤2.6	96 (23.1)	16 (21.9)	80 (23.4)	0.786	56 (24.7)	40 (21.3)	0.415
>2.6	319 (76.9)	57 (78.1)	262 (76.6)		171 (75.3)	148 (78.7)	
LVI							
Absent	339 (81.7)	56 (76.7)	283 (82.7)	0.226	184 (81.1)	155 (82.4)	0.716
Present	76 (18.3)	17 (23.3)	59 (17.3)		43 (18.9)	33 (17.6)	
Nerve invasion							
Absent	329 (79.3)	54 (74.0)	275 (80.4)	0.218	178 (78.4)	151 (80.3)	0.634
Present	86 (20.7)	19 (26.0)	67 (19.6)		49 (21.6)	37 (19.7)	
Histological grade							
Poor	51 (12.3)	11 (15.1)	40 (11.7)	0.827	30 (13.2)	21 (11.2)	0.438
Moderate	302 (72.8)	50 (68.5)	252 (73.7)		157 (69.2)	145 (77.1)	
Well	62 (14.9)	12 (16.4)	50 (14.6)		40 (17.6)	22 (11.7)	
Stage							
I	109 (26.3)	13 (17.8)	96 (28.1)	**0.031**	65 (28.6)	44 (23.4)	0.619
II	109 (26.3)	19 (26.0)	90 (26.3)		55 (24.2)	54 (28.7)	
III	157 (37.8)	30 (41.1)	127 (37.1)		84 (37.0)	73 (38.8)	
IV	40 (9.6)	11 (15.1)	29 (8.5)		23 (10.1)	17 (9.0)	
Tumour							
T1	38 (9.2)	4 (5.5)	34 (9.9)	0.509	25 (11.0)	13 (6.9)	0.077
T2	94 (22.7)	15 (20.5)	79 (23.1)		57 (25.1)	37 (19.7)	
T3	241 (58.1)	49 (67.1)	192 (56.1)		122 (53.7)	119 (63.3)	
T4	42 (10.1)	5 (6.8)	37 (10.8)		23 (10.1)	19 (10.1)	
Nodes							
N0	232 (55.9)	35 (47.9)	197 (57.6)	0.159	127 (55.9)	105 (55.9)	0.823
N1	109 (26.3)	23 (31.5)	86 (25.1)		62 (27.3)	47 (25.0)	
N2	74 (17.8)	15 (20.5)	59 (17.3)		38 (16.7)	36 (19.1)	
Metastasis							
M0	377 (90.8)	62 (84.9)	315 (92.1)	0.054	204 (89.9)	173 (92.0)	0.450
M1	38 (9.2)	11 (15.1)	27 (7.9)		23 (10.1)	15 (8.0)	
Previous abdominal surgery							
No	362 (87.2)	64 (87.7)	298 (87.1)	0.901	199 (87.7)	163 (86.7)	0.770
Yes	53 (12.8)	9 (12.3)	44 (12.9)		28 (12.3)	25 (13.3)	
Any co‐morbidities							
No	302 (72.8)	59 (80.8)	243 (71.1)	0.089	180 (79.3)	122 (64.9)	**0.001**
Yes	113 (27.2)	14 (19.2)	99 (28.9)		47 (20.7)	66 (35.1)	
CEA (ng/mL)							
≤11.6	344 (82.9)	56 (76.7)	288 (84.2)	0.559	194 (85.5)	150 (79.8)	0.126
>11.6	71 (17.1)	17 (23.3)	54 (15.8)		33 (14.5)	38 (20.2)	
CA19‐9 (kU/L)							
≤53.2	373 (89.9)	62 (84.9)	311 (90.9)	0.123	208 (91.6)	165 (87.8)	0.194
>53.2	42 (10.1)	11 (15.1)	31 (9.1)		19 (8.4)	23 (12.2)	
CA125 (U/mL)							
≤15.9	345 (83.1)	63 (86.3)	282 (82.5)	0.426	189 (83.3)	156 (83.0)	0.939
>15.9	70 (16.9)	10 (13.7)	60 (17.5)		38 (16.7)	32 (17.0)	
CA72‐4 (U/mL)							
≤9.4	367 (88.4)	62 (84.9)	305 (89.2)	0.303	196 (86.3)	171 (91.0)	0.143
>9.4	48 (11.6)	11 (15.1)	37 (10.8)		31 (13.7)	17 (9.0)	

Bold was used to highlight values that were statistically significant (*P* < 0.05).

Any co‐morbidities, including cardiovascular disease, cerebrovascular disease, COPD, and diabetes; BMI, body mass index (weight [kg]/height [m^2^]); CA19‐9; CA125; CA72‐4, carbohydrate antigen; CEA, carcino‐embryonic antigen; COPD, chronic obstructive pulmonary disease; IMFA, intermuscular fat area; LVI, lymphovascular invasion.

At the umbilical level, 55.0% and 32.0% of male and female patients, respectively, were in the high IMFA group. Most patients were older and male in the high IMFA group than in the low group (39.4% vs. 19.8%; 70.2% vs. 47.6%, both *P* < 0.001). More patients had a BMI of ≥25 kg/m^2^ (22.3%) in the high IMFA group than in the low group (all *P* < 0.05).

### Factors associated with postoperative complications

At the L3 level, 15 (34.9%) and 53 (14.2%) of patients in the low and high SMD groups, respectively, received blood transfusions during surgery (Table S2). More patients had a shorter LOS in the high IMFA group than in the low IMFA group (81.6% vs. 71.2%, *P* = 0.046, Table S3). More patients received blood transfusions and did not have a colostomy during surgery in the low SMI group than in the high SMI group (28.3% vs. 13.0%, *P* < 0.001; 72.8% vs. 61.0%, *P* = 0.037, Table S4). At the umbilical level, patients with primary anastomosis had lower SMDs than those without (37.3% vs. 26.2%, *P* = 0.044).

At the L3 level, age ≥65 years, tumour diameter >2.6 cm, any preoperative co‐morbidities, preoperative cardiovascular disease, preoperative COPD and LOS > 17 days were risk factors for postoperative complications in the logistic regression analyses (all *P* < 0.05). Correlations between body compositions indices and postoperative complications were not identified (Table S5). Similar results were obtained at the umbilical level (Table S6).

### Factors associated with long‐term survival

Cox multivariate regression analyses were performed to identify correlations between body composition indices and long‐term survival. At the L3 level, Cox multivariate regression analysis revealed that high SMD (HR: 0.357, 95% CI: 0.191–0.665, *P* = 0.001; HR: 0.571, 95% CI: 0.329–0.993, *P* = 0.047) and high SMI (HR: 0.435, 95% CI: 0.254–0.747, *P* = 0.003; HR: 0.568, 95% CI: 0.359–0.897, *P* = 0.015) were significantly associated with better OS and DFS, respectively. TNM stages III and IV (HR: 4.300, 95% CI: 1.805–10.245, *P* = 0.001 and HR: 5.572, 95% CI: 2.094–14.824, *P* = 0.001, respectively), primary anastomosis (HR: 0.435, 95% CI: 0.246–0.770, *P* = 0.004), and CA72–4 (HR: 3.901, 95% CI: 2.284–6.663, *P* < 0.001) were independently associated with OS (Table 3). Nerve invasion (HR: 1.673, 95% CI: 1.055–2.652, *P* = 0.029), TNM stages III and IV (HR: 3.398, 95% CI: 1.652–6.986, *P* = 0.001 and HR: 9.427, 95% CI: 4.190–21.212, *P* < 0.001, respectively), primary anastomosis (HR: 0.543, 95% CI: 0.355–0.831, *P* = 0.005), any postoperative complications (HR: 1.993, 95% CI: 1.140–3.485, *P* = 0.016), CA125 (HR: 1.730, 95% CI: 1.108–2.700, *P* = 0.016), and CA72–4 (HR: 2.289, 95% CI: 1.392–3.764, *P* = 0.001) were independent prognostic factors for DFS in rectal cancer patients (Table S7).

**Table 3:** Univariate and multivariate analysis of factors associated with overall survival at the L3 level

Variables	Univariate analysis		Multivariate analysis	
	HR (95% CI)	*P*	HR (95% CI)	*P*
Sex				
Male	1			
Female	0.773 (0.49–1.220)	0.269		
Age (years)				
>65	1		1	
≥65	2.243 (1.441–3.492)	**<0.001**	1.603 (0.926–2.776)	0.092
BMI (kg/m^2^)				
>25	1			
≥25	0.917 (0.515–1.633)	0.769		
Obstruction before surgery				
Absent	1		1	
Present	2.411 (1.107–5.251)	**0.027**	0.710 (0.273–1.851)	0.484
Family history				
No	1			
Yes	0.857 (0.314–2.343)	0.764		
Radiotherapy				
No	1			
Yes	0.709 (0.259–1.938)	0.503		
Chemotherapy				
No	1			
Yes	0.649 (0.418–1.008)	0.054		
Neoadjuvant treatment				
No	1			
Yes	1.150 (0.500–2.645)	0.742		
Tumour size (cm)				
≤2.6	1		1	
>2.6	2.229 (1.149–4.324)	**0.018**	1.285 (0.632–2.614)	0.489
LVI				
Absent	1		1	
Present	2.347 (1.452–3.794)	**<0.001**	1.262 (0.718–2.219)	0.419
Nerve invasion				
Absent	1		1	
Present	2.473 (1.552–3.940)	**<0.001**	1.140 (0.668–1.944)	0.632
Histological grade				
Poor	1			
Moderate	0.866 (0.455–1.649)	0.662		
Well	0.733 (0.311–1.727)	0.478		
Stage				
I	1		1	
II	1.371 (0.522–3.602)	0.522	1.354 (0.489–3.749)	0.560
III	5.053 (2.281–11.193)	**<0.001**	4.300 (1.805–10.245)	**0.001**
IV	8.284 (3.434–19.986)	**<0.001**	5.572 (2.094–14.824)	**0.001**
Previous abdominal surgery				
No	1		1	
Yes	2.046 (1.197–3.498)	**0.009**	0.991 (0.517–1.899)	0.978
Any co‐morbidities				
No	1			
Yes	1.419 (0.893–2.256)	0.139		
Type of surgery				
Laparoscopy	1			
Laparotomy	0.975 (0.625–1.519)	0.910		
Blood transfusion				
No	1		1	
Yes	1.697 (1.014–2.840)	**0.044**	0.774 (0.413–1.449)	0.423
Primary anastomosis				
No	1		1	
Yes	0.320 (0.206–0.496)	**<0.001**	0.435 (0.246–0.770)	**0.004**
Colostomy				
No	1		1	
Yes	0.532 (0.318–0.891)	**0.016**	0.843 (0.440–1.615)	0.606
Length of stay (days)				
≤17	1			
>17	1.551 (0.949–2.535)	0.080		
Any postoperative complications				
No	1			
Yes	1.205 (0.638–2.278)	0.566		
CEA (ng/mL)				
≤11.6	1		1	
>11.6	3.108 (1.953–4.946)	**<0.001**	1.367 (0.778–2.404)	0.277
CA19‐9 (kU/L)				
≤53.2	1		1	
>53.2	3.246 (1.897–5.556)	**<0.001**	1.551 (0.843–2.854)	0.159
CA125 (U/mL)				
≤15.9	1		1	
>15.9	2.179 (1.323–3.589)	**0.002**	1.369 (0.795–2.356)	0.257
CA72‐4 (U/mL)				
≤9.4	1		1	
>9.4	4.591 (2.854–7.386)	**<0.001**	3.901 (2.284–6.663)	**<0.001**
VFA				
Low	1			
High	1.079 (0.692–1.680)	0.738		
IMFA				
Low	1			
High	1.494 (0.770–2.899)	0.235		
SMA				
Low	1			
High	0.912 (0.584–1.423)	0.685		
SFA				
Low	1			
High	0.914 (0.583–1.433)	0.694		
SMD				
Low	1		1	
High	0.225 (0.136–0.371)	**<0.001**	0.357 (0.191–0.665)	**0.001**
SMI				
Low	1		1	
High	0.482 (0.271–0.858)	**0.013**	0.435 (0.254–0.747)	**0.003**

Bold was used to highlight values that were statistically significant (*P* < 0.05).

Any co‐morbidities, including cardiovascular disease, cerebrovascular disease, COPD, and diabetes; Any postoperative complications, including obstruction, anastomotic fistula, local infection, thrombosis, cardio‐cerebrovascular disease; BMI, body mass index (weight [kg]/height [m^2^]); CA19‐9; CA125; CA72‐4, carbohydrate antigen; CEA, carcino‐embryonic antigen; COPD, chronic obstructive pulmonary disease; IMFA, intermuscular fat area; LVI, lymphovascular invasion; SFA, subcutaneous fat area; SMA, skeletal muscle area; SMD, skeletal muscle density; SMI, skeletal muscle index; VFA, visceral fat area.

At the umbilical level, univariate regression analysis showed that the IMFA (HR: 2.148, *P* = 0.001), SMA (HR: 0.552, *P* = 0.009), SMD (HR: 0.349, *P* < 0.001), and SMI (HR: 0.428, *P* < 0.001) were associated with OS. Cox multivariate regression analysis identified high IMFA (HR: 1.904, 95% CI: 1.068–3.395, *P* = 0.029; HR: 2.064, 95% CI: 1.299–3.280, *P* = 0.002) as an independent prognostic factor for worse OS and DFS, while high SMI (HR: 0.261, 96% CI: 0.132–0.517, *P* < 0.001; HR: 0.595, 95% CI: 0.387–0.913, *P* = 0.018) was an independent prognostic factor for better OS and DFS. TNM stages III and IV (HR: 4.301, 95% CI: 1.797–10.292, *P* = 0.001 and HR: 7.340, 95% CI: 2.734–19.707, *P* < 0.001, respectively), primary anastomosis (HR: 0.321, 95% CI: 0.177–0.580, *P* < 0.001) and CA72–4 (HR: 3.895, 95% CI: 2.214–6.854, *P* < 0.001) were independently associated with OS (Table 4). TNM stages III and IV (HR: 3.402, 95% CI: 1.644–7.041, *P* = 0.001 and HR: 10.768, 95% CI: 4.739–24.463, *P* < 0.001, respectively), primary anastomosis (HR: 0.444, 95% CI: 0.290–0.679, *P* < 0.001), any postoperative complications (HR: 1.955, 95% CI: 1.103–3.464, *P* = 0.022), CA125 (HR: 1.821, 95% CI: 1.163–2.851, *P* = 0.009), and CA72–4 (HR: 2.782, 95% CI: 1.662–4.656, *P* < 0.001) were independently associated with DFS (Table S8).

**Table 4:** Univariate and multivariate analysis of factors associated with overall survival at the umbilical level

Variables	Univariate analysis		Multivariate analysis	
	HR (95% CI)	*P*	HR (95% CI)	*P*
Sex				
Male	1			
Female	0.773 (0.49–1.220)	0.269		
Age (years)				
>65	1		1	
≥65	2.243 (1.441–3.492)	**<0.001**	1.422 (0.819–2.471)	0.211
BMI (kg/m^2^)				
>25	1			
≥25	0.917 (0.515–1.633)	0.769		
Obstruction before surgery				
Absent	1		1	
Present	2.411 (1.107–5.251)	**0.027**	1.286 (0.502–3.296)	0.600
Family history				
No	1			
Yes	0.857 (0.314–2.343)	0.764		
Radiotherapy				
No	1			
Yes	0.709 (0.259–1.938)	0.503		
Chemotherapy				
No	1			
Yes	0.649 (0.418–1.008)	0.054		
Neoadjuvant treatment				
No	1			
Yes	1.150 (0.500–2.645)	0.742		
Tumour size (cm)				
≤2.6	1		1	
>2.6	2.229 (1.149–4.324)	**0.018**	1.086 (0.525–2.246)	0.824
LVI				
Absent	1		1	
Present	2.347 (1.452–3.794)	**<0.001**	1.330 (0.739–2.392)	0.342
Nerve invasion				
Absent	1		1	
Present	2.473 (1.552–3.940)	**<0.001**	1.069 (0.583–1.963)	0.829
Histological grade				
Poor	1			
Moderate	0.866 (0.455–1.649)	0.662		
Well	0.733 (0.311–1.727)	0.478		
Stage				
I	1		1	
II	1.371 (0.522–3.602)	0.522	1.375 (0.499–4.785)	0.538
III	5.053 (2.281–11.193)	**<0.001**	4.301 (1.797–10.292)	**0.001**
IV	8.284 (3.434–19.986)	**<0.001**	7.340 (2.734–19.707)	**<0.001**
Previous abdominal surgery				
No	1		1	
Yes	2.046 (1.197–3.498)	**0.009**	1.545 (0.756–3.158)	0.233
Any co‐morbidities				
No	1			
Yes	1.419 (0.893–2.256)	0.139		
Type of surgery				
Laparoscopy	1			
Laparotomy	0.975 (0.625–1.519)	0.910		
Blood transfusion				
No	1		1	
Yes	1.697 (1.014–2.840)	**0.044**	1.067 (0.583–1.954)	0.834
Primary anastomosis				
No	1		1	
Yes	0.320 (0.206–0.496)	**<0.001**	0.321 (0.177–0.580)	**<0.001**
Colostomy				
No	1		1	
Yes	0.532 (0.318–0.891)	**0.016**	1.001 (0.518–1.934)	0.998
Length of stay (days)				
≤17	1			
>17	1.551 (0.949–2.535)	0.080		
Any postoperative complications				
No	1			
Yes	1.205 (0.638–2.278)	0.566		
CEA (ng/mL)				
≤11.6	1		1	
>11.6	3.108 (1.953–4.946)	**<0.001**	1.324 (0.759–2.308)	0.323
CA19–9 (kU/L)				
≤53.2	1		1	
>53.2	3.246 (1.897–5.556)	**<0.001**	1.854 (0.976–3.523)	0.059
CA125 (U/mL)				
≤15.9	1		1	
>15.9	2.179 (1.323–3.589)	**0.002**	1.464 (0.841–2.548)	0.178
CA72–4 (U/mL)				
≤9.4	1		1	
>9.4	4.591 (2.854–7.386)	**<0.001**	3.895 (2.214–6.854)	**<0.001**
VFA				
Low	1			
High	1.004 (0.636–1.583)	0.988		
IMFA				
Low	1		1	
High	2.148 (1.366–3.380)	**0.001**	1.904 (1.068–3.395)	**0.029**
SMA				
Low	1		1	
High	0.552 (0.353–0.864)	**0.009**	1.651 (0.846–3.221)	0.142
SFA				
Low	1			
High	1.062 (0.683–1.652)	0.788		
SMD				
Low	1		1	
High	0.349 (0.222–0.547)	**<0.001**	0.847 (0.466–1.540)	0.587
SMI				
Low	1		1	
High	0.428 (0.276–0.664)	**<0.001**	0.261 (0.132–0.517)	**<0.001**

Bold was used to highlight values that were statistically significant (*P* < 0.05).

Any co‐morbidities, including cardiovascular disease, cerebrovascular disease, COPD, and diabetes; Any postoperative complications, including obstruction, anastomotic fistula, local infection, thrombosis, cardio‐cerebrovascular disease; BMI, body mass index (weight [kg]/height [m^2^]); CA19‐9; CA125; CA72‐4, carbohydrate antigen; CEA, carcino‐embryonic antigen; COPD, chronic obstructive pulmonary disease; IMFA, intermuscular fat area; LVI, lymphovascular invasion; SFA, subcutaneous fat area; SMA, skeletal muscle area; SMD, skeletal muscle density; SMI, skeletal muscle index; VFA, visceral fat area.

**Figure 2:**
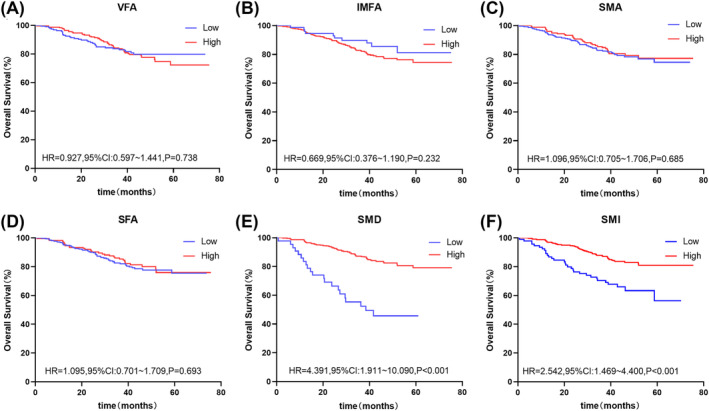
Kaplan–Meier survival curves for overall survival at the L3 level. The Kaplan–Meier survival curves for overall survival grouped by low and high (A) VFA, (B) IMFA, (C) SMA, (D) SFA, (E) SMD, and (F) SMI values at the L3 level. IMFA, intermuscular fat area; L3, lumbar 3 vertebra; SFA, subcutaneous fat area; SMA, skeletal muscle area; SMD, skeletal muscle density; SMI, skeletal muscle index; VFA, visceral fat area.

**Figure 3:**
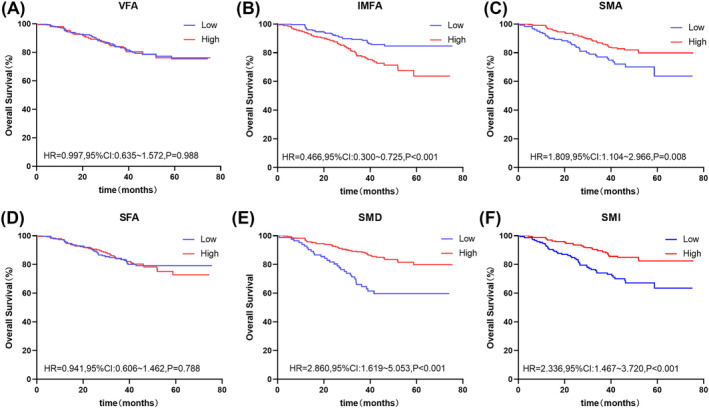
Kaplan–Meier survival curves for overall survival at the umbilical level. The Kaplan–Meier survival curves for overall survival grouped by low and high (A) VFA, (B) IMFA, (C) SMA, (D) SFA, (E) SMD, and (F) SMI values at the umbilical level. IMFA, intermuscular fat area; SFA, subcutaneous fat area; SMA, skeletal muscle area; SMD, skeletal muscle density; SMI, skeletal muscle index; VFA, visceral fat area.

At the L3 level, the Kaplan–Meier curves demonstrated that patients with low SMD (HR: 4.391, 95% CI: 1.911–10.090, *P* < 0.001) and low SMI (HR: 2.542, 95% CI: 1.469–4.400, *P* < 0.001) had worse OS than the high groups. At the umbilical level, patients with low SMA (HR: 1.809, 95% CI: 1.104–2.966, *P* = 0.008), low SMD (HR: 2.860, 95% CI: 1.619–5.053, *P* < 0.001), and low SMI (HR: 2.336, 95% CI: 1.467–3.720, *P* < 0.001) had worse OS than the high groups. Those with low IMFA (HR: 0.466, 95% CI: 0.300–0.725, *P* < 0.001) had better OS than those with high IMFA (Figures 2 and 3). Figures S4 and S5 present the DFS results, which were similar at the L3 and umbilical levels.

### Body composition and clinical indicators combinations for predicting long‐term survival

**Figure 4:**
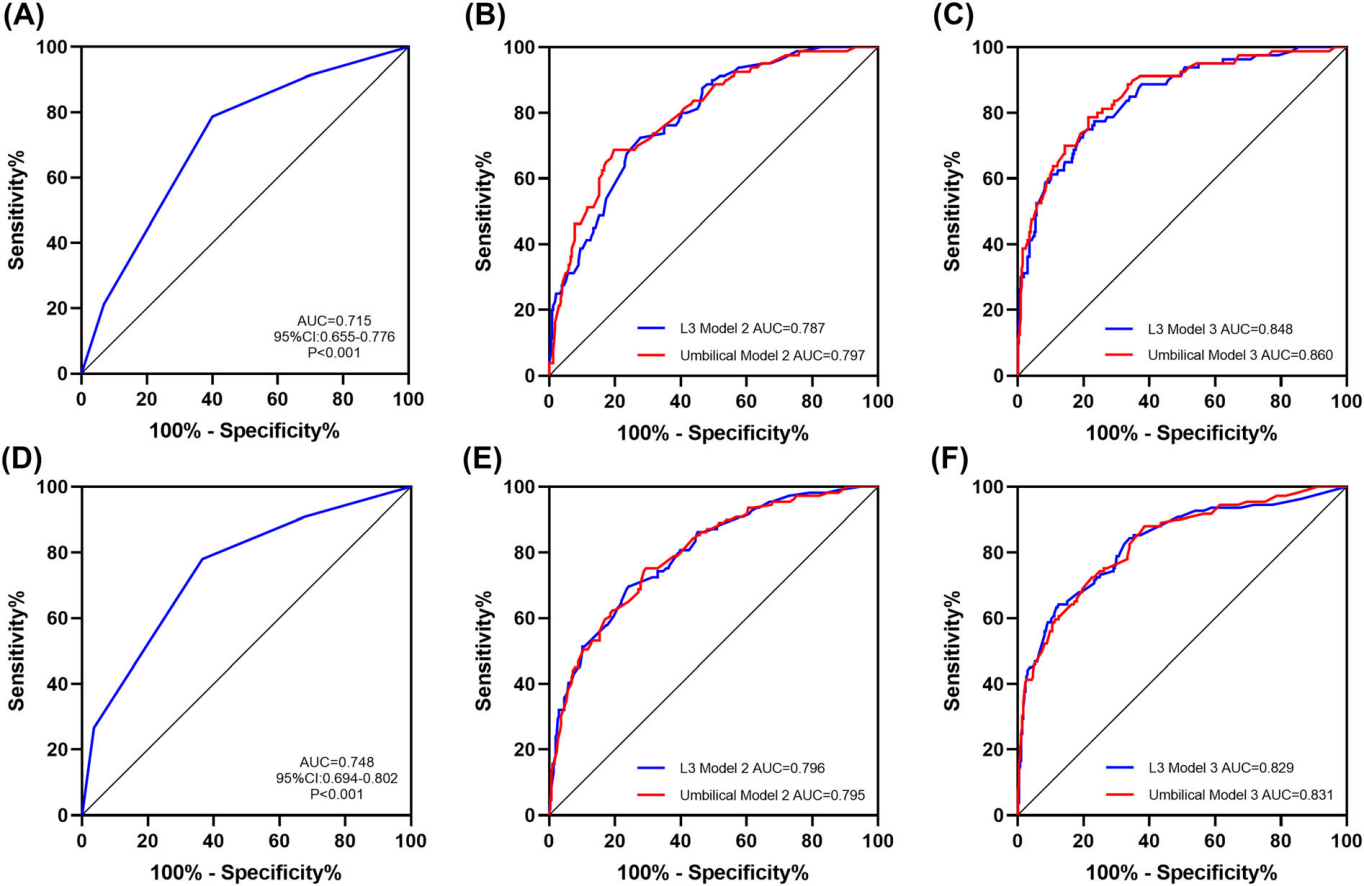
Areas under the receiver operating characteristic curves for overall survival and disease‐free survival. Model 1: TNM stage. Model 2: TNM stage and VFA, IMFA, SMA, SFA, SMD and SMI. Model 3: TNM stage and independent prognostic factors (body composition and clinical indicators). (A–C) AUCs of the models for predicting overall survival. (D–F) The AUCs of models for predicting disease‐free survival. AUC, area under the curve; IMFA, intermuscular fat area; SFA, subcutaneous fat area; SMA, skeletal muscle area; SMD, skeletal muscle density; SMI, skeletal muscle index; TNM, tumour, node, metastasis; VFA, visceral fat area.

The ROC curve analyses demonstrated the predictive abilities of body composition and clinical indicator combinations for predicting long‐term survival (Figure 4). Model 1 (univariate model; TNM stage) had an AUC of 0.715 (95% CI: 0.655–0.776), and Model 2 (TNM stage and six body composition indices at the L3 or umbilical levels) had AUCs of 0.787 (L3 level) and 0.797 (umbilical level) for predicting OS. Model 3 (TNM stage and independent prognostic indicators at the L3 or umbilical levels) had AUCs of 0.848 (L3 level) and 0.860 (umbilical level) for predicting OS. The multivariate models incorporating body composition and clinical indicators outperformed the univariate TNM stage model. Moreover, calibration curves assessing these models indicated good predictive accuracy between the models' actual and predicted probabilities (Figure S6).

## Discussion

This study identified significant associations between better long‐term survival and body composition (high SMD and high SMI at the L3 level, low IMFA and high SMI at the umbilical level). We then constructed a novel prognostic model for patients with rectal cancer by combining the preoperative body composition and clinical indicators. This model had a better predictive ability for OS and DFS than the traditional TNM stage, making it a valuable tool for risk assessment.

Sarcopenia and myosteatosis affect the long‐term prognosis of other cancers, such as oesophageal cancer,^23^ head and neck cancer,^24^ malignant lymphoma,^25^ and pancreatic cancer.^26^ We found low SMI and low SMD were associated with worse OS and DFS at the L3 level in patients with rectal cancer, consistent with the results of previous studies.^11^, ^12^, ^27^, ^28^ However, a recent study reported that reduced muscle density in patients with CRC significantly affects long‐term survival, but survival did not correlated with decreased muscle mass.^29^ Van Vugt et al.^30^ found that skeletal muscle mass and density could not independently predict the long‐term outcomes of patients with CRC. These inconsistent results may be attributed to differences in race, cut‐off values, and the selection of clinical characteristics.

Our study also analysed umbilical‐level images, finding a significant association between long‐term survival and IMFA instead of SMD, perhaps because the body composition distribution differed between the two levels, resulting in different cut‐off values and groupings. Low SMD and high IMFA levels share the same intermuscular and intramuscular fat infiltration mechanisms, leading to myosteatosis. Our findings indicate that IMFA is an important indicator worthy of further research and discussion.

The European Working Group on Sarcopenia in Older People updated the definition of sarcopenia in 2019, stating that low muscle strength, low muscle quantity/quality, or low physical performance could be diagnosed as sarcopenia.^31^ In CT images, sarcopenia manifests as decreased skeletal muscle mass, but the mechanisms affecting prognosis remain unclear. Sarcopenia affects physical mobility and causes metabolic dysfunctions, such as energy homeostasis, heat regulation, insulin sensitivity, and amino acid metabolism.^32^ Moreover, increased inter‐ and intramuscular fat infiltration often accompanies muscle depletion.^33^ In CT images, this manifests as decreased skeletal muscle density and infiltration of intermuscular fat tissue. Myosteaotosis also negatively correlates with the systemic inflammatory response, especially with the neutrophil‐to‐lymphocyte ratio and modified Glasgow Prognostic Score.^34^, ^35^ Innate immune cells, such as neutrophils and monocytes/macrophages, promote systemic inflammation, which can suppress cytotoxic immunity and potentially cause tumour progression.^36^, ^37^ Furthermore, intra‐ and intermuscular fat deposition is associated with insulin resistance, loss of strength and motor dysfunction, which can cause contractile impairment and metabolic and endocrine abnormalities, ultimately resulting in cancer cachexia.^38^, ^39^ CT‐quantified skeletal muscle indices may provide a new approach for identifying cancer patients with a poor prognosis, and requires further investigation.

Obesity is another factor that affects the long‐term survival of patients with CRC, mainly including subcutaneous fat and visceral fat, but studies have reported inconsistent results. Jin‐Mok et al.^9^ found a high SFA was an independent prognostic factor for improving the CRC prognosis, and VFA was associated with long‐term survival. Conversely, Benoit et al.^10^ found that neither VFA nor SFA was significantly correlated with rectal cancer prognosis. These inconsistent results suggest a nonlinear association between abdominal adiposity and long‐term survival in patients with CRC. A recent study demonstrated that visceral adipose tissue was a prognostic factor for mortality in a reverse L‐shaped pattern, whereas subcutaneous adipose tissue showed a J‐shaped pattern.^40^ Further research is needed to determine the optimal cut‐off value for adipose tissue.

Previous studies have investigated the correlations between body composition and CRC surgery complications. Malietzis et al.^11^ found that the presence of myosteatosis was associated with a prolonged LOS, whereas decreased skeletal muscle mass was associated with an increased risk of 30‐day morbidity and mortality. Jeroen et al.^30^ demonstrated that decreased skeletal muscle mass and density were predictive indicators of postoperative complications, mortality, LOS, and discharge status in patients undergoing curative resection for CRC. Our study found that CT‐quantified muscle and fat indices were not associated with postoperative complications. The low incidence of postoperative complications in our study cohort could be the primary reason for this result. However, Arayne et al.^41^ recently investigated the relationship between sarcopenia and postoperative complications in patients with rectal cancer and found no association, supporting our results. Most previous studies have focused on CRC, but obvious differences in molecular carcinogenesis, pathology, embryological origin, metastatic patterns, and surgical approaches exist between colon and rectal cancers,^42^, ^43^ which could also explain the inconsistent conclusions. Future research should investigate colon and rectal cancer separately.

We also developed univariate and multivariate predictive models for postoperative OS and DFS. The multivariate models at the umbilical level performed well (AUCs: 0.797 and 0.860 for OS), suggesting that future studies should not be limited to the L3 level. These results also confirmed the correlation between CT‐quantified body composition and rectal cancer prognosis, which might assist clinicians in predicting long‐term survival in rectal cancer patients. However, the sample size should be expanded in future studies to improve the models' generalizability and accuracy.

This study has two limitations. First, a nonlinear relationship may exist between abdominal fat and long‐term survival in patients with rectal cancer, we did not conduct further subgroup analyses. Second, this was a single‐centre retrospective study, which resulted in a low incidence of postoperative complications in our study cohort. Multicenter prospective clinical studies should be performed in the future to validate the feasibility of our model.

This study evaluated the associations between preoperative body composition and postoperative complications in patients with rectal cancer and their value in long‐term survival and recurrence. Including body composition indicators in the prediction model can significantly improve the model's predictive performance for patient prognosis. These results could help identify patients with a poor prognosis and assisting clinicians in providing personalized management and treatment.

## Funding

This study was supported by the National Key Research and Development Program of China (2023YFC2413500 to CZ), the National Nature Science Foundation of China (82302313 to FW), the China Postdoctoral Science Foundation (2023M731216 to YC), and the Jianghan Talent Funding (02.05.22030036 to YC).

## Conflict of interest

The authors declare no potential conflicts of interest.

## Supporting information


**Figure S1.** Flowchart of the population.

**Figure S2.** Optimal cut‐off values based on overall survival at the lumbar 3 vertebra level.

**Figure S3.** Optimal cut‐off values based on overall survival at the umbilical level.

**Figure S4.** Kaplan–Meier survival curves for disease‐free survival at the L3 level.

**Figure S5.** Kaplan–Meier survival curves for disease‐free survival at the umbilical level.

**Figure S6.** Calibration curves of predictive models.

**Table S1.** Patient characteristics based on SMI.

**Table S2.** Patient management and postoperative complications based on SMD.

**Table S3.** Patient management and postoperative complications based on IMFA.

**Table S4.** Patient management and postoperative complications based on SMI.

**Table S5.** Logistic regression analysis for postoperative complications at the L3 level.

**Table S6.** Logistic regression analysis for postoperative complications at the umbilical level.

**Table S7.** Univariate and multivariate analysis of factors associated with disease‐free survival at the L3 level.

**Table S8.** Univariate and multivariate analysis of factors associated with disease‐free survival at the umbilical level.
