# Supplementary figures and images for: Influence of skeletal muscle and intermuscular fat on postoperative complications and long‐term survival in rectal cancer patients
Source: J Cachexia Sarcopenia Muscle. 2024 Jan 31;15(2):702–17. doi: 10.1002/jcsm.13424 (PMC10995272; doi:10.1002/jcsm.13424)

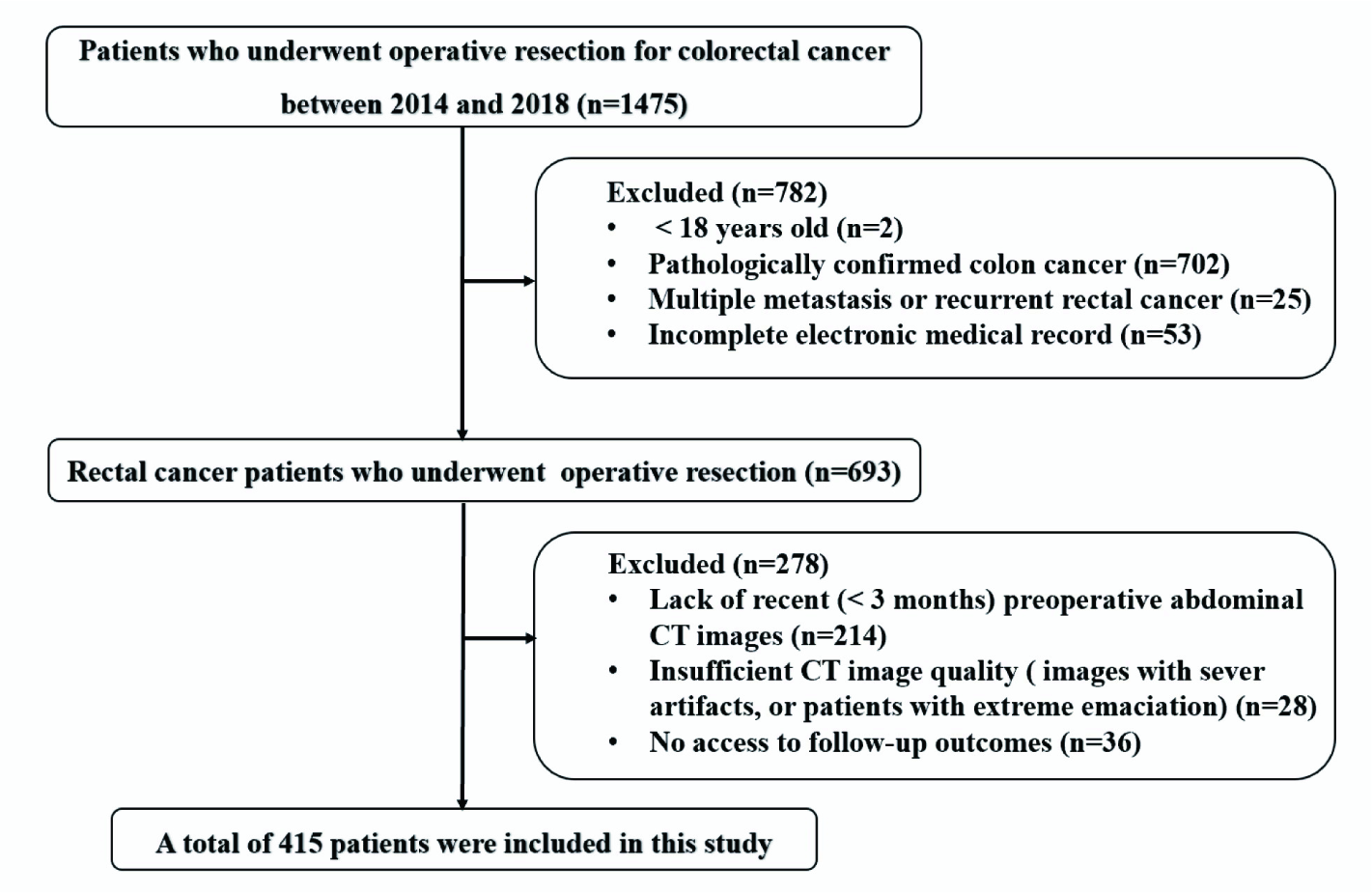

Supplement: Supplementary file 1 — Figure S1. Flowchart of the population. [file JCSM-15-702-s007.tif]

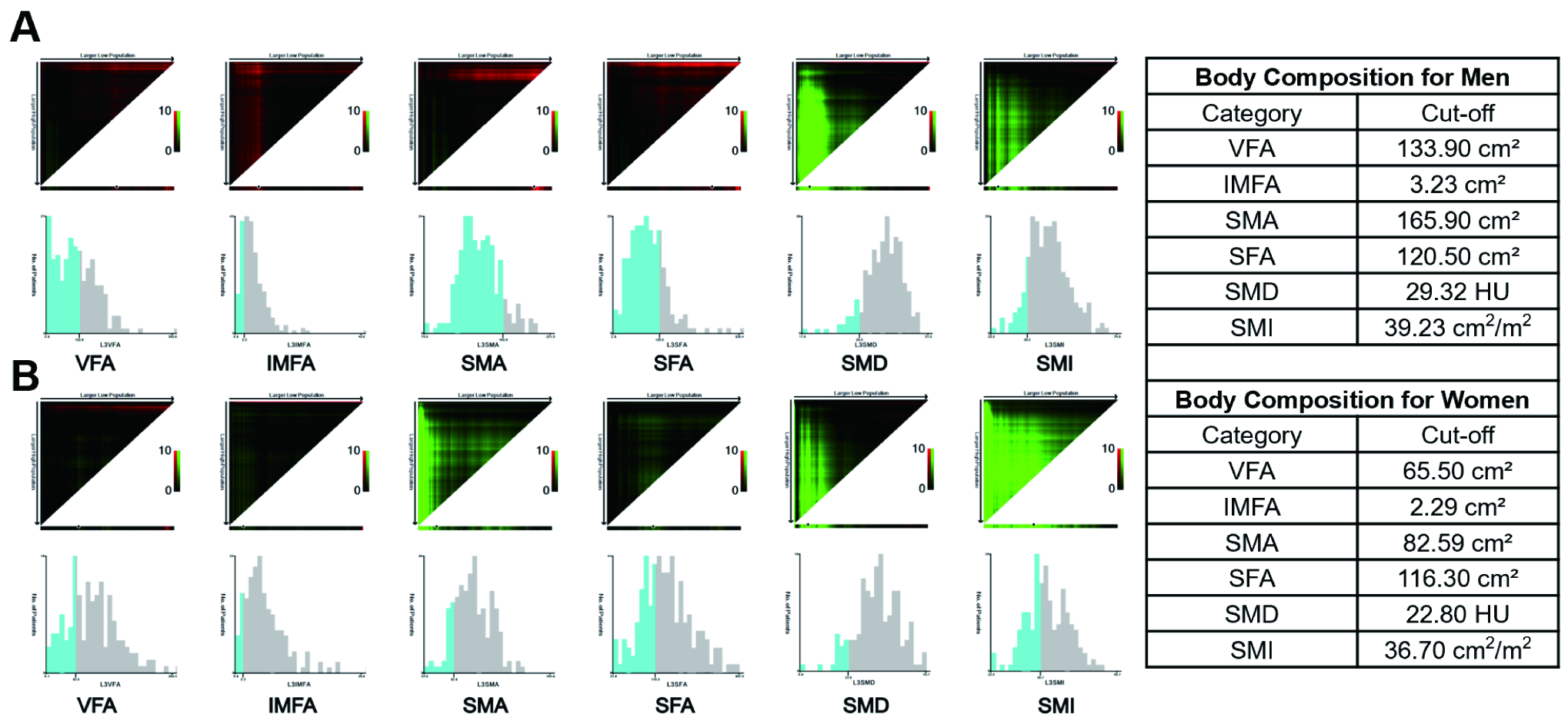

Supplement: Supplementary file 2 — Figure S2. Optimal cut‐off values based on overall survival at the lumbar 3 vertebra level. [file JCSM-15-702-s012.tif]

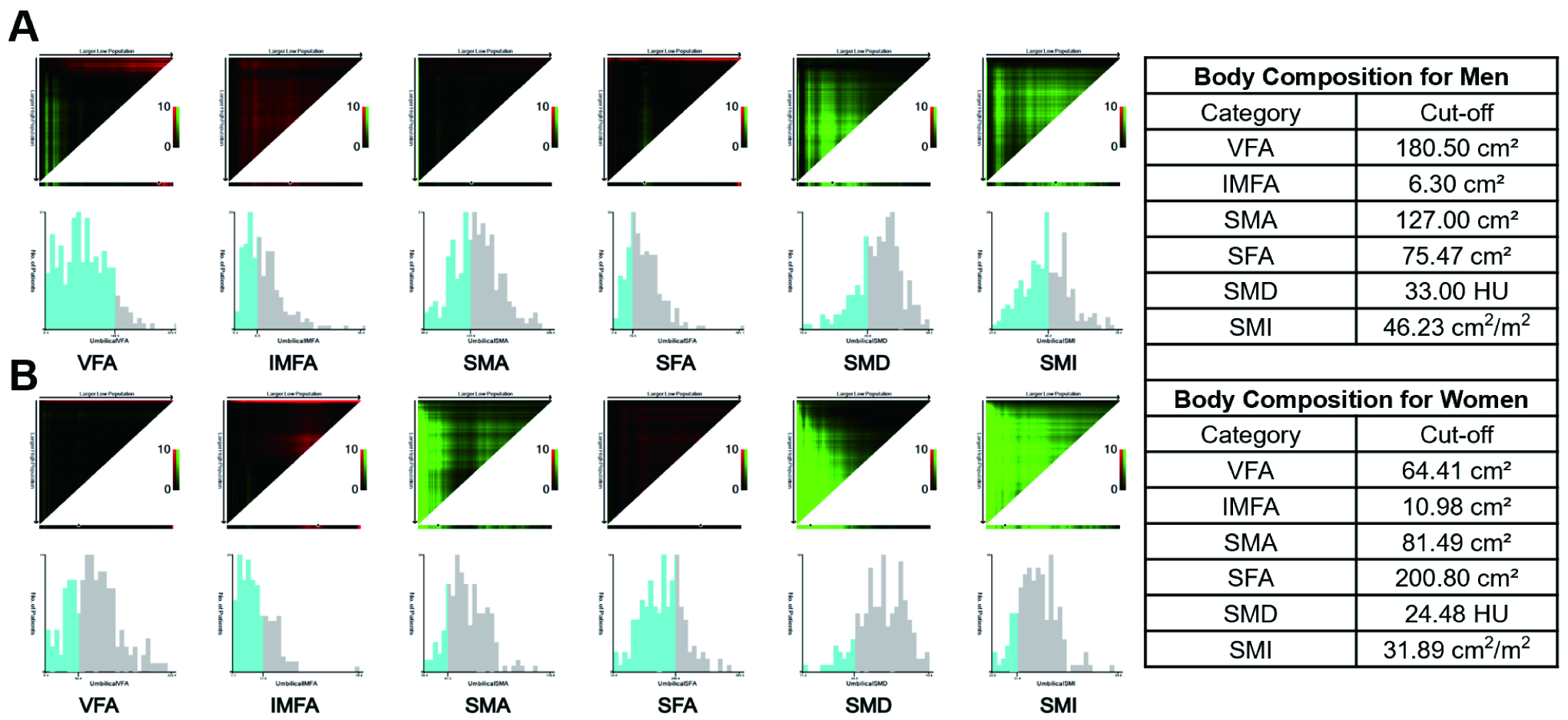

Supplement: Supplementary file 3 — Figure S3. Optimal cut‐off values based on overall survival at the umbilical level. [file JCSM-15-702-s006.tif]

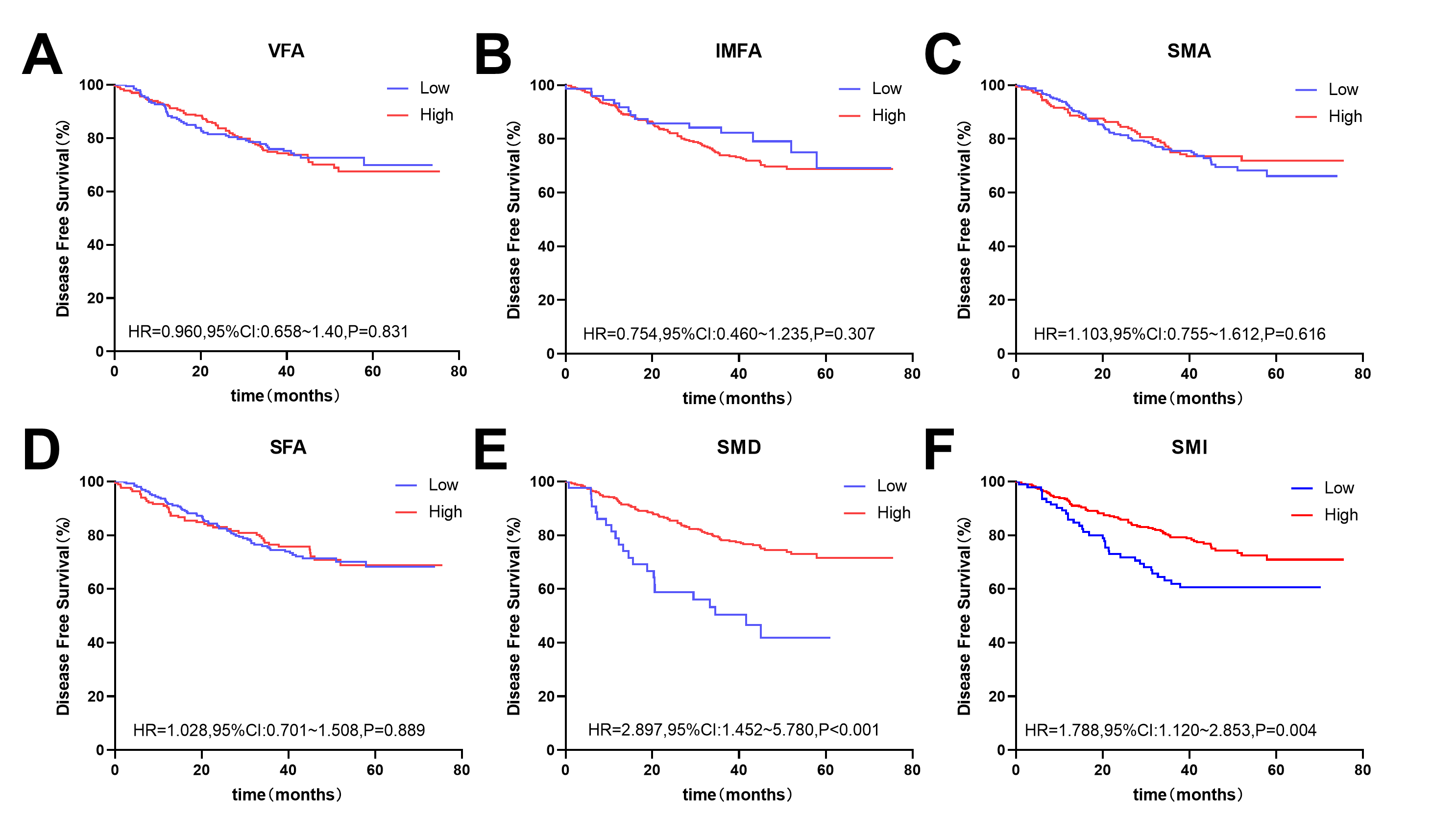

Supplement: Supplementary file 4 — Figure S4. Kaplan–Meier survival curves for disease‐free survival at the L3 level. [file JCSM-15-702-s004.tif]

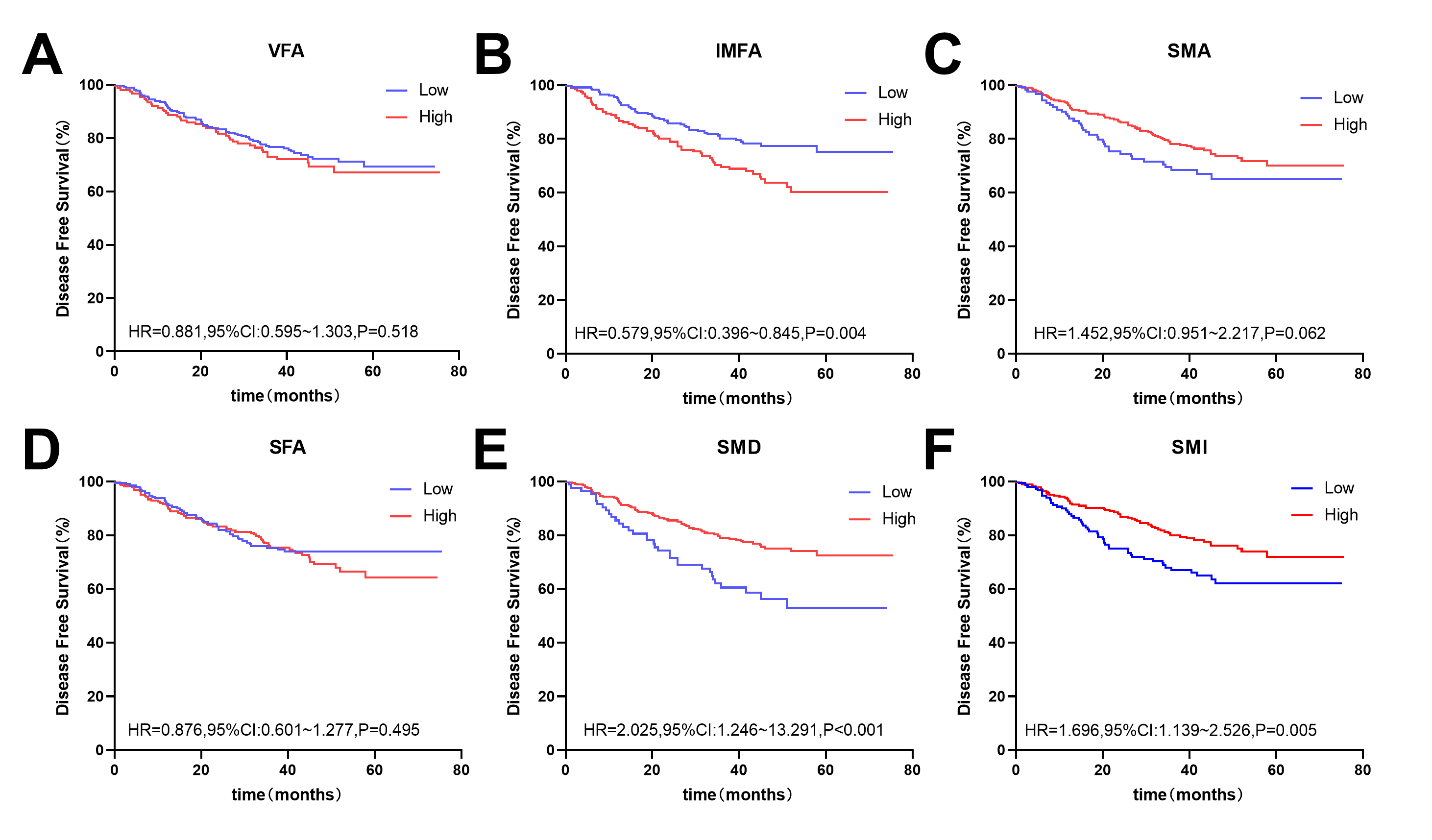

Supplement: Supplementary file 5 — Figure S5. Kaplan–Meier survival curves for disease‐free survival at the umbilical level. [file JCSM-15-702-s010.tif]
