# Supplementary material for: Influence of skeletal muscle and intermuscular fat on postoperative complications and long‐term survival in rectal cancer patients
Source: J Cachexia Sarcopenia Muscle. 2024 Jan 31;15(2):702–17. doi: 10.1002/jcsm.13424 (PMC10995272; doi:10.1002/jcsm.13424)
Supplement: Supplementary file 7 — Table S1. Patient characteristics based on SMI. [file JCSM-15-702-s009.docx]

**Table S1 Patient characteristics based on SMI**

| **Characteristics** | | **N (%)** |  |  |  |  |  |  |  |  |  |
| --- | --- | --- | --- | --- | --- | --- | --- | --- | --- | --- | --- |
|  |  | **Overall (N=415)** |  | **L3 SMI** | | **P** |  | **Umbilical SMI** | | **P** |  |
|  |  |  |  | **Low (n=92)** | **High (n=323)** |  |  | **Low (n=148)** | **High (n=267)** |  |  |
| Sex | |  |  |  |  |  |  |  |  |  |  |
|  | Male | 240 (57.8) |  | 26 (28.3) | 214 (66.3) | **<0.001** |  | 124 (83.8) | 116 (43.4) | **<0.001** |  |
|  | Female | 175 (42.2) |  | 66 (71.7) | 109 (33.7) |  |  | 24 (16.2) | 151 (56.6) |  |  |
| Age (years) | |  |  |  |  |  |  |  |  |  |  |
|  | <65 | 296 (71.3) |  | 52 (56.5) | 244 (75.5) | **<0.001** |  | 81 (54.7) | 215 (80.5) | **<0.001** |  |
|  | ≥65 | 119 (28.7) |  | 40 (43.5) | 79 (24.5) |  |  | 67 (45.3) | 52 (19.5) |  |  |
| BMI (kg/m²) | |  |  |  |  |  |  |  |  |  |  |
|  | <25 | 341 (82.2) |  | 86 (93.5) | 255 (78.9) | **0.001** |  | 135 (91.2) | 206 (77.2) | **<0.001** |  |
|  | ≥25 | 74 (17.8) |  | 6 (6.5) | 68 (21.1) |  |  | 13 (8.8) | 61 (22.8) |  |  |
| Obstruction before surgery | | |  |  |  |  |  |  |  |  |  |
|  | Absent | 394 (94.9) |  | 84 (91.3) | 310 (96.0) | 0.071 |  | 137 (92.6) | 257 (96.3) | 0.101 |  |
|  | Present | 21 (5.1) |  | 8 (8.7) | 13 (4.0) |  |  | 11 (7.4) | 10 (3.7) |  |  |
| Family history | |  |  |  |  |  |  |  |  |  |  |
|  | No | 392 (94.5) |  | 85 (92.4) | 307 (95.0) | 0.326 |  | 140 (94.6) | 252 (94.4) | 0.928 |  |
|  | Yes | 23 (5.5) |  | 7 (7.6) | 16 (5.0) |  |  | 8 (5.4) | 15 (5.6) |  |  |
| Radiotherapy | |  |  |  |  |  |  |  |  |  |  |
|  | No | 386 (93.0) |  | 89 (96.7) | 297 (92.0) | 0.112 |  | 135 (91.2) | 251 (94.0) | 0.285 |  |
|  | Yes | 29 (7.0) |  | 3 (3.3) | 26 (8.0) |  |  | 13 (8.8) | 16 (6.0) |  |  |
| Chemotherapy | |  |  |  |  |  |  |  |  |  |  |
|  | No | 168 (40.5) |  | 44 (47.8) | 124 (38.4) | 0.104 |  | 67 (45.3) | 101 (37.8) | 0.139 |  |
|  | Yes | 247 (59.5) |  | 48 (52.2) | 199 (61.6) |  |  | 81 (54.7) | 166 (62.2) |  |  |
| Neoadjuvant treatment | | |  |  |  |  |  |  |  |  |  |
|  | No | 383 (92.3) |  | 83 (90.2) | 300 (92.9) | 0.398 |  | 140 (94.6) | 243 (91.0) | 0.190 |  |
|  | Yes | 32 (7.7) |  | 9 (9.8) | 23 (7.1) |  |  | 8 (5.4) | 24 (9.0) |  |  |
| Tumor size (cm) | |  |  |  |  |  |  |  |  |  |  |
|  | ≤2.6 | 96 (23.1) |  | 22 (23.9) | 74 (22.9) | 0.841 |  | 31 (20.9) | 65 (24.3) | 0.432 |  |
|  | >2.6 | 319 (76.9) |  | 70 (76.1) | 249 (77.1) |  |  | 117 (79.1) | 202 (75.7) |  |  |
| LVI | |  |  |  |  |  |  |  |  |  |  |
|  | Absent | 339 (81.7) |  | 80 (87.0) | 259 (80.2) | 0.139 |  | 123 (83.1) | 216 (80.9) | 0.577 |  |
|  | Present | 76 (18.3) |  | 12 (13.0) | 64 (19.8) |  |  | 25 (16.9) | 51 (19.1) |  |  |
| Nerve invasion | |  |  |  |  |  |  |  |  |  |  |
|  | Absent | 329 (79.3) |  | 76 (82.6) | 253 (78.3) | 0.372 |  | 115 (77.7) | 214 (80.1) | 0.556 |  |
|  | Present | 86 (20.7) |  | 16 (17.4) | 70 (21.7) |  |  | 33 (22.3) | 53 (19.9) |  |  |
| Histological grade | |  |  |  |  |  |  |  |  |  |  |
|  | Poor | 51 (12.3) |  | 6 (6.5) | 45 (13.9) | 0.312 |  | 20 (13.5) | 31 (11.6) | 0.077 |  |
|  | Moderate | 302 (72.8) |  | 73 (79.3) | 229 (70.9) |  |  | 113 (76.4) | 189 (70.8) |  |  |
|  | Well | 62 (14.9) |  | 13 (14.1) | 49 (15.2) |  |  | 15 (10.1) | 47 (17.6) |  |  |
| Stage | |  |  |  |  |  |  |  |  |  |  |
|  | I | 109 (26.3) |  | 28 (30.4) | 81 (25.1) | 0.540 |  | 39 (26.4) | 70 (26.2) | 0.717 |  |
|  | II | 109 (26.3) |  | 21 (22.8) | 88 (27.2) |  |  | 40 (27.0) | 69 (25.8) |  |  |
|  | III | 157 (37.8) |  | 35 (38.0) | 122 (37.8) |  |  | 57 (38.5) | 100 (37.5) |  |  |
|  | IV | 40 (9.6) |  | 8 (8.7) | 32 (9.9) |  |  | 12 (8.1) | 28 (10.5) |  |  |
| Tumor | |  |  |  |  |  |  |  |  |  |  |
|  | T1 | 38 (9.2) |  | 11 (12.0) | 27 (8.4) | 0.992 |  | 12 (8.1) | 26 (9.7) | 0.479 |  |
|  | T2 | 94 (22.7) |  | 22 (23.9) | 72 (22.3) |  |  | 33 (22.3) | 61 (22.8) |  |  |
|  | T3 | 241 (58.1) |  | 44 (47.8) | 197 (61.0) |  |  | 86 (58.1) | 155 (58.1) |  |  |
|  | T4 | 42 (10.1) |  | 15 (16.3) | 27 (8.4) |  |  | 17 (11.5) | 25 (9.4) |  |  |
| Nodes | |  |  |  |  |  |  |  |  |  |  |
|  | N0 | 232 (55.9) |  | 52 (56.5) | 180 (55.7) | 0.883 |  | 84 (56.8) | 148 (55.4) | 0.879 |  |
|  | N1 | 109 (26.3) |  | 24 (26.1) | 85 (26.3) |  |  | 37 (25.0) | 72 (27.0) |  |  |
|  | N2 | 74 (17.8) |  | 16 (17.4) | 58 (18.0) |  |  | 27 (18.2) | 47 (17.6) |  |  |
| Metastasis | |  |  |  |  |  |  |  |  |  |  |
|  | M0 | 377 (90.8) |  | 85 (92.4) | 292 (90.4) | 0.560 |  | 137 (92.6) | 240 (89.9) | 0.365 |  |
|  | M1 | 38 (9.2) |  | 7 (7.6) | 31 (9.6) |  |  | 11 (7.4) | 27 (10.1) |  |  |
| Previous abdominal surgery | | |  |  |  |  |  |  |  |  |  |
|  | No | 362 (87.2) |  | 70 (76.1) | 292 (90.4) | **<0.001** |  | 135 (91.2) | 227 (85.0) | 0.070 |  |
|  | Yes | 53 (12.8) |  | 22 (23.9) | 31 (9.6) |  |  | 13 (8.8) | 40 (15.0) |  |  |
| Any comorbidities | |  |  |  |  |  |  |  |  |  |  |
|  | No | 302 (72.8) |  | 70 (76.1) | 232 (71.8) | 0.418 |  | 103 (69.6) | 199 (74.5) | 0.279 |  |
|  | Yes | 113 (27.2) |  | 22 (23.9) | 91 (28.2) |  |  | 45 (30.4) | 68 (25.5) |  |  |
| CEA (ng/mL) | |  |  |  |  |  |  |  |  |  |  |
|  | ≤11.6 | 344 (82.9) |  | 78 (84.8) | 266 (82.4) | 0.585 |  | 120 (81.1) | 224 (83.9) | 0.466 |  |
|  | >11.6 | 71 (17.1) |  | 14 (15.2) | 57 (17.6) |  |  | 28 (18.9) | 43 (16.1) |  |  |
| CA19-9 (kU/L) | |  |  |  |  |  |  |  |  |  |  |
|  | ≤53.2 | 373 (89.9) |  | 78 (84.8) | 295 (91.3) | 0.066 |  | 136 (91.9) | 237 (88.8) | 0.312 |  |
|  | >53.2 | 42 (10.1) |  | 14 (15.2) | 28 (8.7) |  |  | 12 (8.1) | 30 (11.2) |  |  |
| CA125 (U/mL) | |  |  |  |  |  |  |  |  |  |  |
|  | ≤15.9 | 345 (83.1) |  | 68 (73.9) | 277 (85.8) | **0.007** |  | 121 (81.8) | 224 (83.9) | 0.577 |  |
|  | >15.9 | 70 (16.9) |  | 24 (26.1) | 46 (14.2) |  |  | 27 (18.2) | 43 (16.1) |  |  |
| CA72-4 (U/mL) | |  |  |  |  |  |  |  |  |  |  |
|  | ≤9.4 | 367 (88.4) |  | 80 (87.0) | 287 (88.9) | 0.616 |  | 130 (87.8) | 237 (88.8) | 0.777 |  |
|  | >9.4 | 48 (11.6) |  | 12 (13.0) | 36 (11.1) |  |  | 18 (12.2) | 30 (11.2) |  |  |
| **Abbreviations: SMI, skeletal muscle index. BMI, body mass index (weight [kg]/height [m^2^]); LVI, lymphovascular invasion; COPD, chronic obstructive pulmonary disease; CEA, carcino-embryonic antigen; CA19-9; CA125; CA72-4, carbohydrate antigen; Any comorbidities, including cardiovascular disease, cerebrovascular disease, COPD, and diabetes.** | | | | | | | | | | |  |
|  |  |  |  |  |  |  |  |  |  |  |  |
| **Bold was used to highlight values that were statistically significant (P<0.05).** | | | | | | | | | | |  |
