# Supplementary material for: Influence of skeletal muscle and intermuscular fat on postoperative complications and long‐term survival in rectal cancer patients
Source: J Cachexia Sarcopenia Muscle. 2024 Jan 31;15(2):702–17. doi: 10.1002/jcsm.13424 (PMC10995272; doi:10.1002/jcsm.13424)
Supplement: Supplementary file 8 — Table S2. Patient management and postoperative complications based on SMD. [file JCSM-15-702-s001.docx]

**Table S2 Patient management and postoperative complications based on SMD**

| **Characteristics** | | **N (%)** |  |  |  |  |  |  |  |  |
| --- | --- | --- | --- | --- | --- | --- | --- | --- | --- | --- |
|  |  | **Overall (N=415)** |  | **L3 SMD** | | **P** |  | **Umbilical SMD** | | **P** |
|  |  |  |  | **Low (n=43)** | **High (n=372)** |  |  | **Low (n=83)** | **High (n=332)** |  |
| Type of surgery | |  |  |  |  |  |  |  |  |  |
|  | Laparoscopy | 179 (43.1) |  | 17 (39.5) | 162 (43.5) | 0.615 |  | 32 (38.6) | 147 (44.3) | 0.346 |
|  | Laparotomy | 236 (56.9) |  | 26 (60.5) | 210 (56.5) |  |  | 51 (61.4) | 185 (55.7) |  |
| Blood transfusion | |  |  |  |  |  |  |  |  |  |
|  | No | 347 (83.6) |  | 28 (65.1) | 319 (85.8) | **0.001** |  | 65 (78.3) | 282 (84.9) | 0.145 |
|  | Yes | 68 (16.4) |  | 15 (34.9) | 53 (14.2) |  |  | 18 (21.7) | 50 (15.1) |  |
| Primary anastomosis | |  |  |  |  |  |  |  |  |  |
|  | No | 118 (28.4) |  | 16 (37.2) | 102 (27.4) | 0.178 |  | 31 (37.3) | 87 (26.2) | **0.044** |
|  | Yes | 297 (71.6) |  | 27 (62.8) | 270 (72.6) |  |  | 52 (62.7) | 245 (73.8) |  |
| Colostomy | |  |  |  |  |  |  |  |  |  |
|  | No | 264 (63.6) |  | 26 (60.5) | 238 (64.0) | 0.650 |  | 57 (68.7) | 207 (62.3) | 0.284 |
|  | Yes | 151 (36.4) |  | 17 (39.5) | 134 (36.0) |  |  | 26 (31.3) | 125 (37.7) |  |
| Length of stay (days) | |  |  |  |  |  |  |  |  |  |
|  | ≤17 | 331 (79.8) |  | 37 (86.0) | 294 (79.0) | 0.278 |  | 66 (79.5) | 265 (79.8) | 0.951 |
|  | >17 | 84 (20.2) |  | 6 (14.0) | 78 (21.0) |  |  | 17 (20.5) | 67 (20.2) |  |
| Postoperative complications | |  |  |  |  |  |  |  |  |  |
| Total patients | |  |  |  |  |  |  |  |  |  |
|  | No | 365 (88.0) |  | 35 (81.4) | 330 (88.7) | 0.163 |  | 72 (86.7) | 293 (88.3) | 0.706 |
|  | Yes | 50 (12.0) |  | 8 (18.6) | 42 (11.3) |  |  | 11 (13.3) | 39 (11.7) |  |
| Obstruction | |  |  |  |  |  |  |  |  |  |
|  | No | 409 (98.6) |  | 41 (95.3) | 368 (98.9) | 0.236 |  | 82 (98.8) | 327 (98.5) | 1.000 |
|  | Yes | 6 (1.4) |  | 2 (4.7) | 4 (1.1) |  |  | 1 (1.2) | 5 (1.5) |  |
| Anastomotic fistula | |  |  |  |  |  |  |  |  |  |
|  | No | 394 (94.9) |  | 39 (90.7) | 355 (95.4) | 0.331 |  | 77 (92.8) | 317 (95.5) | 0.467 |
|  | Yes | 21 (5.1) |  | 4 (9.3) | 17 (4.6) |  |  | 6 (7.2) | 15 (4.5) |  |
| Local infection | |  |  |  |  |  |  |  |  |  |
|  | No | 379 (91.3) |  | 38 (88.4) | 341 (91.7) | 0.660 |  | 75 (90.4) | 304 (91.6) | 0.727 |
|  | Yes | 36 (8.7) |  | 5 (11.6) | 31 (8.3) |  |  | 8 (9.6) | 28 (8.4) |  |
| Thrombosis | |  |  |  |  |  |  |  |  |  |
|  | No | 408 (98.3) |  | 42 (97.7) | 366 (98.4) | 1.000 |  | 83 (100.0) | 325 (97.9) | 0.391 |
|  | Yes | 7 (1.7) |  | 1 (2.3) | 6 (1.6) |  |  | 0 (0.0) | 7 (2.1) |  |
| Cardio-cerebrovascular disease | |  |  |  |  |  |  |  |  |  |
|  | No | 412 (99.3) |  | 42 (97.7) | 370 (99.5) | 0.280 |  | 82 (98.8) | 330 (99.4) | 0.489 |
|  | Yes | 3 (0.7) |  | 1 (2.3) | 2 (0.5) |  |  | 1 (1.2) | 2 (0.6) |  |
| **Abbreviations: SMD, skeletal muscle density.** | | | | | | | | | | |
| **Bold was used to highlight values that were statistically significant (P<0.05).** | | | | | | | | | | |
