# Supplementary material for: Influence of skeletal muscle and intermuscular fat on postoperative complications and long‐term survival in rectal cancer patients
Source: J Cachexia Sarcopenia Muscle. 2024 Jan 31;15(2):702–17. doi: 10.1002/jcsm.13424 (PMC10995272; doi:10.1002/jcsm.13424)
Supplement: Supplementary file 9 — Table S3. Patient management and postoperative complications based on IMFA. [file JCSM-15-702-s005.docx]

**Table S3 Patient management and postoperative complications based on IMFA**

| **Characteristics** | | **N (%)** |  |  |  |  |  |  |  |  |
| --- | --- | --- | --- | --- | --- | --- | --- | --- | --- | --- |
|  |  | **Overall (N=415)** |  | **L3 IMFA** | | **P** |  | **Umbilical IMFA** | | **P** |
|  |  |  |  | **Low (n=73)** | **High (n=342)** |  |  | **Low (n=277)** | **High (n=188)** |  |
| Type of surgery | |  |  |  |  |  |  |  |  |  |
|  | Laparoscopy | 179 (43.1) |  | 33 (45.2) | 146 (42.7) | 0.694 |  | 102 (36.8) | 77 (41.0) | 0.416 |
|  | Laparotomy | 236 (56.9) |  | 40 (54.8) | 196 (57.3) |  |  | 125 (45.1) | 111 (59.0) |  |
| Blood transfusion | |  |  |  |  |  |  |  |  |  |
|  | No | 347 (83.6) |  | 66 (90.4) | 281 (82.2) | 0.084 |  | 191 (69.0) | 156 (83.0) | 0.750 |
|  | Yes | 68 (16.4) |  | 7 (9.6) | 61 (17.8) |  |  | 36 (13.0) | 32 (17.0) |  |
| Primary anastomosis | |  |  |  |  |  |  |  |  |  |
|  | No | 118 (28.4) |  | 22 (30.1) | 96 (28.1) | 0.722 |  | 66 (23.8) | 52 (27.7) | 0.750 |
|  | Yes | 297 (71.6) |  | 51 (69.9) | 246 (71.9) |  |  | 161 (58.1) | 136 (72.3) |  |
| Colostomy | |  |  |  |  |  |  |  |  |  |
|  | No | 264 (63.6) |  | 48 (65.8) | 216 (63.2) | 0.676 |  | 146 (52.7) | 118 (62.8) | 0.744 |
|  | Yes | 151 (36.4) |  | 25 (34.2) | 126 (36.8) |  |  | 81 (29.2) | 70 (37.2) |  |
| Length of stay (days) | |  |  |  |  |  |  |  |  |  |
|  | ≤17 | 331 (79.8) |  | 52 (71.2) | 279 (81.6) | **0.046** |  | 182 (65.7) | 149 (79.3) | 0.816 |
|  | >17 | 84 (20.2) |  | 21 (28.8) | 63 (18.4) |  |  | 45 (16.2) | 39 (20.7) |  |
| Postoperative complications | | |  |  |  |  |  |  |  |  |
| Total patients | |  |  |  |  |  |  |  |  |  |
|  | No | 365 (88.0) |  | 64 (87.7) | 301 (88.0) | 0.935 |  | 201 (72.6) | 164 (87.2) | 0.683 |
|  | Yes | 50 (12.0) |  | 9 (12.3) | 41 (12.0) |  |  | 26 (9.4) | 24 (12.8) |  |
| Obstruction | |  |  |  |  |  |  |  |  |  |
|  | No | 409 (98.6) |  | 72 (98.6) | 337 (98.5) | 0.715 |  | 225 (81.2) | 184 (97.9) | 0.417 |
|  | Yes | 6 (1.4) |  | 1 (1.4) | 5 (1.5) |  |  | 2 (0.7) | 4 (2.1) |  |
| Anastomotic fistula | |  |  |  |  |  |  |  |  |  |
|  | No | 394 (94.9) |  | 70 (95.9) | 324 (94.7) | 0.909 |  | 218 (78.7) | 176 (93.6) | 0.263 |
|  | Yes | 21 (5.1) |  | 3 (4.1) | 18 (5.3) |  |  | 9 (3.2) | 12 (6.4) |  |
| Local infection | |  |  |  |  |  |  |  |  |  |
|  | No | 379 (91.3) |  | 66 (90.4) | 313 (91.5) | 0.760 |  | 208 (75.1) | 171 (91.0) | 0.809 |
|  | Yes | 36 (8.7) |  | 7 (9.6) | 29 (8.5) |  |  | 19 (6.9) | 17 (9.0) |  |
| Thrombosis | |  |  |  |  |  |  |  |  |  |
|  | No | 408 (98.3) |  | 72 (98.6) | 336 (98.2) | 1.000 |  | 222 (80.1) | 186 (98.9) | 0.464 |
|  | Yes | 7 (1.7) |  | 1 (1.4) | 6 (1.8) |  |  | 5 (1.8) | 2 (1.1) |  |
| Cardio-cerebrovascular disease | | |  |  |  |  |  |  |  |  |
|  | No | 412 (99.3) |  | 73 (100.0) | 339 (99.1) | 1.000 |  | 226 (81.6) | 186 (98.9) | 0.592 |
|  | Yes | 3 (0.7) |  | 0 (0.0) | 3 (0.9) |  |  | 1 (0.4) | 2 (1.1) |  |
| **Abbreviations: IMFA, intermuscular fat area.** | | | | | | | | | | |
| **Bold was used to highlight values that were statistically significant (P<0.05).** | | | | | | | | | | |
