# Supplementary material for: Influence of skeletal muscle and intermuscular fat on postoperative complications and long‐term survival in rectal cancer patients
Source: J Cachexia Sarcopenia Muscle. 2024 Jan 31;15(2):702–17. doi: 10.1002/jcsm.13424 (PMC10995272; doi:10.1002/jcsm.13424)
Supplement: Supplementary file 10 — Table S4. Patient management and postoperative complications based on SMI. [file JCSM-15-702-s008.docx]

**Table S4 Patient management and postoperative complications based on SMI**

| **Characteristics** | | **N (%)** |  |  |  |  |  |  |  |  |
| --- | --- | --- | --- | --- | --- | --- | --- | --- | --- | --- |
|  |  | **Overall (N=415)** |  | **L3 SMI** | | **P** |  | **Umbilical SMI** | | **P** |
|  |  |  |  | **Low (n=92)** | **High (n=323)** |  |  | **Low (n=148)** | **High (n=267)** |  |
|  |  |  |  |  |  |  |  |  |  |  |
|  | Laparoscopy | 179 (43.1) |  | 44 (47.8) | 135 (41.8) | 0.303 |  | 67 (45.3) | 112 (41.9) | 0.513 |
|  | Laparotomy | 236 (56.9) |  | 48 (52.2) | 188 (58.2) |  |  | 81 (54.7) | 155 (58.1) |  |
| Blood transfusion | |  |  |  |  |  |  |  |  |  |
|  | No | 347 (83.6) |  | 66 (71.7) | 281 (87.0) | **<0.001** |  | 122 (82.4) | 225 (84.3) | 0.628 |
|  | Yes | 68 (16.4) |  | 26 (28.3) | 42 (13.0) |  |  | 26 (17.6) | 42 (15.7) |  |
| Primary anastomosis | |  |  |  |  |  |  |  |  |  |
|  | No | 118 (28.4) |  | 31 (33.7) | 87 (26.9) | 0.205 |  | 41 (27.7) | 77 (28.8) | 0.806 |
|  | Yes | 297 (71.6) |  | 61 (66.3) | 236 (73.1) |  |  | 107 (72.3) | 190 (71.2) |  |
| Colostomy | |  |  |  |  |  |  |  |  |  |
|  | No | 264 (63.6) |  | 67 (72.8) | 197 (61.0) | **0.037** |  | 96 (64.9) | 168 (62.9) | 0.693 |
|  | Yes | 151 (36.4) |  | 25 (27.2) | 126 (39.0) |  |  | 52 (35.1) | 99 (37.1) |  |
| Length of stay (days) | |  |  |  |  |  |  |  |  |  |
|  | ≤17 | 331 (79.8) |  | 75 (81.5) | 256 (79.3) | 0.633 |  | 120 (81.1) | 211 (79.0) | 0.618 |
|  | >17 | 84 (20.2) |  | 17 (18.5) | 67 (20.7) |  |  | 28 (18.9) | 56 (21.0) |  |
| Postoperative complications | |  |  |  |  |  |  |  |  |  |
| Total patients | |  |  |  |  |  |  |  |  |  |
|  | No | 365 (88.0) |  | 85 (92.4) | 280 (86.7) | 0.138 |  | 131 (88.5) | 234 (87.6) | 0.794 |
|  | Yes | 50 (12.0) |  | 7 (7.6) | 43 (13.3) |  |  | 17 (11.5) | 33 (12.4) |  |
| Obstruction | |  |  |  |  |  |  |  |  |  |
|  | No | 409 (98.6) |  | 92 (100.0) | 317 (98.1) | 0.346 |  | 147 (99.3) | 262 (98.1) | 0.428 |
|  | Yes | 6 (1.4) |  | 0 (0.0) | 6 (1.9) |  |  | 1 (0.7) | 5 (1.9) |  |
| Anastomotic fistula | |  |  |  |  |  |  |  |  |  |
|  | No | 394 (94.9) |  | 87 (94.6) | 307 (95.0) | 1.000 |  | 140 (94.6) | 254 (95.1) | 0.811 |
|  | Yes | 21 (5.1) |  | 5 (5.4) | 16 (5.0) |  |  | 8 (5.4) | 13 (4.9) |  |
| Local infection | |  |  |  |  |  |  |  |  |  |
|  | No | 379 (91.3) |  | 85 (92.4) | 294 (91.0) | 0.681 |  | 135 (91.2) | 244 (91.4) | 0.953 |
|  | Yes | 36 (8.7) |  | 7 (7.6) | 29 (9.0) |  |  | 13 (8.8) | 23 (8.6) |  |
| Thrombosis | |  |  |  |  |  |  |  |  |  |
|  | No | 408 (98.3) |  | 92 (100.0) | 316 (97.8) | 0.334 |  | 146 (98.6) | 262 (98.1) | 1.000 |
|  | Yes | 7 (1.7) |  | 0 (0.0) | 7 (2.2) |  |  | 2 (1.4) | 5 (1.9) |  |
| Cardio-cerebrovascular disease | |  |  |  |  |  |  |  |  |  |
|  | No | 412 (99.3) |  | 92 (100.0) | 320 (99.1) | 1.000 |  | 146 (98.6) | 266 (99.6) | 0.290 |
|  | Yes | 3 (0.7) |  | 0 (0.0) | 3 (0.9) |  |  | 2 (1.4) | 1 (0.4) |  |
| **Abbreviations: SMI, skeletal muscle index.** | | | | | | | | | | |
| **Bold was used to highlight values that were statistically significant (P<0.05).** | | | | | | | | | | |
