# Supplementary material for: Influence of skeletal muscle and intermuscular fat on postoperative complications and long‐term survival in rectal cancer patients
Source: J Cachexia Sarcopenia Muscle. 2024 Jan 31;15(2):702–17. doi: 10.1002/jcsm.13424 (PMC10995272; doi:10.1002/jcsm.13424)
Supplement: Supplementary file 11 — Table S5. Logistic regression analysis for postoperative complications at the L3 level. [file JCSM-15-702-s011.docx]

**Table S5 Logistic regression analysis for postoperative complications at the L3 level**

| **Variables** | | **Postoperative complications** | | | |  | **Univariate analysis** | | |  | **Multivariate analysis** | |
| --- | --- | --- | --- | --- | --- | --- | --- | --- | --- | --- | --- | --- |
|  |  | **No (365)** | | **Yes (50)** | |  |  |  |  |  |  |  |
|  |  | **count** | **%** | **count** | **%** |  | **HR (95%Cl)** |  | **P** |  | **HR (95%Cl)** | **P** |
| Sex | |  |  |  |  | |  |  | | |  |  |
|  | Male | 209 | 57.3 | 31 | 62.0 |  | 1 |  | | |  |  |
|  | Female | 156 | 42.7 | 19 | 38.0 |  | 0.821 (0.447~1.508) |  | 0.525 |  |  |  |
| Age (years) | |  |  |  |  |  |  |  |  |  |  |  |
|  | <65 | 268 | 73.4 | 28 | 56.0 |  | 1 |  |  |  | 1 |  |
|  | ≥65 | 97 | 26.6 | 22 | 44.0 |  | 2.171 (1.186~3.974) |  | **0.012** |  | 1.804 (0.902~3.606) | 0.095 |
| BMI (kg/m²) | |  |  |  |  |  |  |  |  |  |  |  |
|  | <25 | 297 | 81.4 | 44 | 88.0 |  | 1 |  |  |  |  |  |
|  | ≥25 | 68 | 18.6 | 6 | 12.0 |  | 0.596 (0.244~1.454) |  | 0.255 |  |  |  |
| Obstruction before surgery | | | |  |  |  |  |  |  |  |  |  |
|  | Absent | 348 | 95.3 | 46 | 92.0 |  | 1 |  |  |  |  |  |
|  | Present | 17 | 4.7 | 4 | 8.0 |  | 1.780 (0.574~5.520) |  | 0.318 |  |  |  |
| Family history | |  |  |  |  |  |  |  |  |  |  |  |
|  | No | 344 | 94.2 | 48 | 96.0 |  | 1 |  |  |  |  |  |
|  | Yes | 21 | 5.8 | 2 | 4.0 |  | 0.683 (0.155~3.003) |  | 0.613 |  |  |  |
| Radiotherapy | |  |  |  |  |  |  |  |  |  |  |  |
|  | No | 339 | 92.9 | 47 | 94.0 |  | 1 |  |  |  |  |  |
|  | Yes | 26 | 7.1 | 3 | 6.0 |  | 0.832 (0.242~2.857) |  | 0.770 |  |  |  |
| Chemotherapy | |  |  |  |  |  |  |  |  |  |  |  |
|  | No | 147 | 40.3 | 21 | 42.0 |  | 1 |  |  |  |  |  |
|  | Yes | 218 | 59.7 | 29 | 58.0 |  | 0.931 (0.511~1.696) |  | 0.816 |  |  |  |
| Neoadjuvant treatment | |  |  |  |  |  |  |  |  |  |  |  |
|  | No | 334 | 91.5 | 49 | 98.0 |  | 1 |  |  |  |  |  |
|  | Yes | 31 | 8.5 | 1 | 2.0 |  | 0.220 (0.029~1.647) |  | 0.140 |  |  |  |
| Tumor size (cm) | |  |  |  |  |  |  |  |  |  |  |  |
|  | ≤2.6 | 93 | 25.5 | 3 | 6.0 |  | 1 |  |  |  | 1 |  |
|  | >2.6 | 272 | 74.5 | 47 | 94.0 |  | 5.357 (1.629~17.619) |  | **0.006** |  | 4.396 (1.279~15.109) | **0.019** |
| LVI | |  |  |  |  |  |  |  |  |  |  |  |
|  | Absent | 299 | 81.9 | 40 | 80.0 |  | 1 |  |  |  |  |  |
|  | Present | 66 | 18.1 | 10 | 20.0 |  | 1.133 (0.539~2.380) |  | 0.742 |  |  |  |
| Nerve invasion | |  |  |  |  |  |  |  |  |  |  |  |
|  | Absent | 293 | 80.3 | 36 | 72.0 |  | 1 |  |  |  |  |  |
|  | Present | 72 | 19.7 | 14 | 28.0 |  | 1.583 (0.811~3.090) |  | 0.179 |  |  |  |
| Histological grade | |  |  |  |  |  |  |  |  |  |  |  |
|  | Poor | 44 | 12.1 | 7 | 14.0 |  | 1 |  |  |  |  |  |
|  | Moderate | 267 | 73.2 | 35 | 70.0 |  | 0.824 (0.345~1.971) |  | 0.663 |  |  |  |
|  | Well | 54 | 14.8 | 8 | 16.0 |  | 0.931 (0.313~2.769) |  | 0.898 |  |  |  |
| Stage |  |  |  |  |  |  |  |  |  |  |  |  |
|  | I | 101 | 27.7 | 8 | 16.0 |  | 1 |  |  |  |  |  |
|  | II | 90 | 24.7 | 19 | 38.0 |  | 2.665 (1.113~6.384) |  | 0.028 |  |  |  |
|  | III | 140 | 38.4 | 17 | 34.0 |  | 1.533 (0.637~3.690) |  | 0.340 |  |  |  |
|  | IV | 34 | 9.3 | 6 | 12.0 |  | 2.228 (0.721~6.880) |  | 0.164 |  |  |  |
| Previous abdominal surgery | | |  |  |  |  |  |  |  |  |  |  |
|  | No | 315 | 86.3 | 47 | 94.0 |  | 1 |  |  |  |  |  |
|  | Yes | 50 | 13.7 | 3 | 6.0 |  | 0.402 (0.121~1.341) |  | 0.138 |  |  |  |
| Comorbidities | |  |  |  |  |  |  |  |  |  |  |  |
| Total patients | |  |  |  |  |  |  |  |  |  |  |  |
|  | No | 274 | 75.1 | 28 | 56.0 |  | 1 |  |  |  | 1 |  |
|  | Yes | 91 | 24.9 | 22 | 44.0 |  | 2.366 (1.290~4.340) |  | **0.005** |  | 1.246 (0.331~4.684) | 0.745 |
| Cardiovascular disease | | |  |  |  |  |  |  |  |  |  |  |
|  | No | 289 | 79.2 | 33 | 66.0 |  | 1 |  |  |  | 1 |  |
|  | Yes | 76 | 20.8 | 17 | 34.0 |  | 1.959 (1.036~3.705） |  | **0.039** |  | 1.984 (0.501~7.857) | 0.329 |
| Cerebrovascular disease | | |  |  |  |  |  |  |  |  |  |  |
|  | No | 362 | 99.2 | 48 | 96.0 |  | 1 |  |  |  |  |  |
|  | Yes | 3 | 0.8 | 2 | 4.0 |  | 5.028 (0.819~30.856) |  | 0.081 |  |  |  |
| COPD |  |  |  |  |  |  |  |  |  |  |  |  |
|  | No | 361 | 98.9 | 47 | 94.0 |  | 1 |  |  |  | 1 |  |
|  | Yes | 4 | 1.1 | 3 | 6.0 |  | 5.761 (1.251~26.537) |  | **0.025** |  | 5.744 (0.866~38.077) | 0.070 |
| Diabetes |  |  |  |  |  |  |  |  |  |  |  |  |
|  | No | 342 | 93.7 | 46 | 92.0 |  | 1 |  |  |  |  |  |
|  | Yes | 23 | 6.3 | 4 | 8.0 |  | 1.293 (0.428~3.906) |  | 0.649 |  |  |  |
| Hematologic disease | |  |  |  |  |  |  |  |  |  |  |  |
|  | No | 363 | 99.5 | 50 | 100.0 |  |  |  |  |  |  |  |
|  | Yes | 2 | 0.5 | 0 | 0.0 |  |  |  |  |  |  |  |
| Type of surgery | |  |  |  |  |  |  |  |  |  |  |  |
|  | Laparoscopy | 159 | 43.6 | 20 | 40.0 |  | 1 |  |  |  |  |  |
|  | Laparotomy | 206 | 56.4 | 30 | 60.0 |  | 1.158 (0.634~2.115) |  | 0.634 |  |  |  |
| Blood transfusion | |  |  |  |  |  |  |  |  |  |  |  |
|  | No | 308 | 84.4 | 39 | 78.0 |  | 1 |  |  |  |  |  |
|  | Yes | 57 | 15.6 | 11 | 22.0 |  | 1.524 (0.737~3.151) |  | 0.256 |  |  |  |
| Primary anastomosis | |  |  |  |  |  |  |  |  |  |  |  |
|  | No | 102 | 27.9 | 16 | 32.0 |  | 1 |  |  |  |  |  |
|  | Yes | 263 | 72.1 | 34 | 68.0 |  | 0.824 (0.436~1.558) |  | 0.552 |  |  |  |
| Colostomy | |  |  |  |  |  |  |  |  |  |  |  |
|  | No | 234 | 64.1 | 30 | 60.0 |  | 1 |  |  |  |  |  |
|  | Yes | 131 | 35.9 | 20 | 40.0 |  | 1.191 (0.650~2.180) |  | 0.571 |  |  |  |
| Length of stay (days) | |  |  |  |  |  |  |  |  |  |  |  |
|  | ≤17 | 309 | 84.7 | 22 | 44.0 |  | 1 |  |  |  | 1 |  |
|  | >17 | 56 | 15.3 | 28 | 56.0 |  | 7.023 (3.752~13.143) |  | **<0.001** |  | 8.388 (4.198~16.760) | **<0.001** |
| CEA (ng/mL) | |  |  |  |  |  |  |  |  |  |  |  |
|  | ≤11.6 | 305 | 83.6 | 39 | 78.0 |  | 1 |  |  |  |  |  |
|  | >11.6 | 60 | 16.4 | 11 | 22.0 |  | 1.434 (0.695~2.958) |  | 0.329 |  |  |  |
| CA19-9 (kU/L) | |  |  |  |  |  |  |  |  |  |  |  |
|  | ≤53.2 | 328 | 89.9 | 45 | 90.0 |  | 1 |  |  |  |  |  |
|  | >53.2 | 37 | 10.1 | 5 | 10.0 |  | 0.985 (0.368~2.636) |  | 0.976 |  |  |  |
| CA125 (U/mL) | |  |  |  |  |  |  |  |  |  |  |  |
|  | ≤15.9 | 303 | 83.0 | 42 | 84.0 |  | 1 |  |  |  |  |  |
|  | >15.9 | 62 | 17.0 | 8 | 16.0 |  | 0.931 (0.417~2.080) |  | 0.861 |  |  |  |
| CA72-4 (U/mL) | |  |  |  |  |  |  |  |  |  |  |  |
|  | ≤9.4 | 322 | 88.2 | 45 | 90.0 |  | 1 |  |  |  |  |  |
|  | >9.4 | 43 | 11.8 | 5 | 10.0 |  | 0.832 (0.313~2.211) |  | 0.712 |  |  |  |
| VFA |  |  |  |  |  |  |  |  |  |  |  |  |
|  | Low | 166 | 45.5 | 23 | 46.0 |  | 1 |  |  |  |  |  |
|  | High | 199 | 54.5 | 27 | 54.0 |  | 0.979 (0.541~1.772) |  | 0.945 |  |  |  |
| IMFA |  |  |  |  |  |  |  |  |  |  |  |  |
|  | Low | 64 | 17.5 | 9 | 18.0 |  | 1 |  |  |  |  |  |
|  | High | 301 | 82.5 | 41 | 82.0 |  | 0.969 (0.448~2.093) |  | 0.935 |  |  |  |
| SMA |  |  |  |  |  |  |  |  |  |  |  |  |
|  | Low | 209 | 57.3 | 29 | 58.0 |  | 1 |  |  |  |  |  |
|  | High | 156 | 42.7 | 21 | 42.0 |  | 0.970 (0.533~1.765) |  | 0.921 |  |  |  |
| SFA |  |  |  |  |  |  |  |  |  |  |  |  |
|  | Low | 219 | 60.0 | 30 | 60.0 |  | 1 |  |  |  |  |  |
|  | High | 146 | 40.0 | 20 | 40.0 |  | 1.000 (0.547~1.828) |  | 1.000 |  |  |  |
| SMD |  |  |  |  |  |  |  |  |  |  |  |  |
|  | Low | 35 | 9.6 | 8 | 16.0 |  | 1 |  |  |  |  |  |
|  | High | 330 | 90.4 | 42 | 84.0 |  | 0.557 (0.242~1.280) |  | 0.168 |  |  |  |
| SMI |  |  |  |  |  |  |  |  |  |  |  |  |
|  | Low | 85 | 23.3 | 7 | 14.0 |  | 1 |  |  |  |  |  |
|  | High | 280 | 76.7 | 43 | 86.0 |  | 1.865 (0.809~4.298) |  | 0.143 |  |  |  |
| **Abbreviations: Postoperative complications, including obstruction, anastomotic fistula, local infection, thrombosis, cardio-cerebrovascular disease; BMI, body mass index (weight [kg]/height [m^2^]); LVI, lymphovascular invasion; COPD, chronic obstructive pulmonary disease; CEA, carcino-embryonic antigen; CA19-9; CA125; CA72-4, carbohydrate antigen; VFA, visceral fat area; IMFA, intermuscular fat area; SMA, skeletal muscle area; SFA, subcutaneous fat area; SMD, skeletal muscle density; SMI, skeletal muscle index.** | | | | | | | | | | | | |
| **Bold was used to highlight values that were statistically significant (P<0.05).** | | | | | | | | | | | | |
