# Supplementary material for: Influence of skeletal muscle and intermuscular fat on postoperative complications and long‐term survival in rectal cancer patients
Source: J Cachexia Sarcopenia Muscle. 2024 Jan 31;15(2):702–17. doi: 10.1002/jcsm.13424 (PMC10995272; doi:10.1002/jcsm.13424)
Supplement: Supplementary file 13 — Table S7. Univariate and multivariate analysis of factors associated with disease‐free survival at the L3 level. [file JCSM-15-702-s014.docx]

**Table S7 Univariate and multivariate analysis of factors associated with disease-free survival at the L3 level**

| **Variables** | | **Univariate analysis** | |  | **Multivariate analysis** | |  |  |
| --- | --- | --- | --- | --- | --- | --- | --- | --- |
|  |  | **HR (95%CI)** | **P** |  | **HR (95%CI)** | **P** |  |  |
| Sex | |  |  |  |  |  |  |  |
|  | Male | 1 |  |  |  |  |  |  |
|  | Female | 0.793(0.537~1.171) | 0.244 |  |  |  |  |  |
| Age (years) | |  |  |  |  |  |  |  |
|  | >65 | 1 |  |  | 1 |  |  |  |
|  | ≥65 | 1.734(1.179~2.551) | **0.005** |  | 1.193 (0.744~1.915) | 0.464 |  |  |
| BMI (kg/m²) | |  |  |  |  |  |  | |
|  | >25 | 1 |  |  |  |  |  |  |
|  | ≥25 | 1.013(0.624~1.646) | 0.957 |  |  |  |  |  |
| Obstruction before surgery | | |  |  |  |  |  | |
|  | Absent | 1 |  |  |  |  |  |  |
|  | Present | 1.417(0.621~3.232) | 0.408 |  |  |  |  |  |
| Family history | |  |  |  |  |  |  | |
|  | No | 1 |  |  |  |  |  |  |
|  | Yes | 1.172(0.545~2.520) | 0.685 |  |  |  |  |  |
| Radiotherapy | |  |  |  |  |  |  | |
|  | No | 1 |  |  |  |  |  |  |
|  | Yes | 0.791(0.347~1.802) | 0.577 |  |  |  |  |  |
| Chemotherapy | |  |  |  |  |  |  | |
|  | No | 1 |  |  |  |  |  |  |
|  | Yes | 0.770(0.528~1.122) | 0.173 |  |  |  |  |  |
| Neoadjuvant treatment | |  |  |  |  |  |  | |
|  | No | 1 |  |  |  |  |  |  |
|  | Yes | 1.146(0.557~2.355) | 0.711 |  |  |  |  |  |
| Tumor size (cm) | |  |  |  |  |  |  | |
|  | ≤2.6 | 1 |  |  | 1 |  |  |  |
|  | >2.6 | 2.090(1.212~3.605) | **0.008** |  | 1.223 (0.685~2.182) | 0.496 |  |  |
| LVI | |  |  |  |  |  |  | |
|  | Absent | 1 |  |  | 1 |  |  |  |
|  | Present | 2.983(2.008~4.432) | **<0.001** |  | 1.319 (0.820~2.120) | 0.254 |  |  |
| Nerve invasion | |  |  |  |  |  |  | |
|  | Absent | 1 |  |  | 1 |  |  |  |
|  | Present | 3.235(2.192~4.775) | **<0.001** |  | 1.673 (1.055~2.652) | **0.029** |  |  |
| Histological grade | |  |  |  |  |  |  | |
|  | Poor | 1 |  |  |  |  |  |  |
|  | Moderate | 0.663(0.397~1.107) | 0.116 |  |  |  |  |  |
|  | Well | 0.533(0.261~1.088) | 0.084 |  |  |  |  |  |
| Stage | |  |  |  |  |  |  | |
|  | I | 1 |  |  | 1 |  |  |  |
|  | II | 1.386(0.616~3.121) | 0.430 |  | 1.154 (0.496~2.685) | 0.740 |  |  |
|  | III | 4.478(2.285~8.778) | **<0.001** |  | 3.398 (1.652~6.986) | **0.001** |  |  |
|  | IV | 13.523(6.575~27.812) | **<0.001** |  | 9.427 (4.190~21.212) | **<0.001** |  |  |
| Previous abdominal surgery | | |  |  |  |  |  | |
|  | No | 1 |  |  |  |  |  |  |
|  | Yes | 1.176(0.682~2.028) | 0.561 |  |  |  |  |  |
| Any comorbidities | |  |  |  |  |  |  | |
|  | No | 1 |  |  |  |  |  |  |
|  | Yes | 1.358(0.911~2.025) | 0.133 |  |  |  |  |  |
| Type of surgery | |  |  |  |  |  |  | |
|  | Laparoscopy | 1 |  |  |  |  |  |  |
|  | Laparotomy | 0.984(0.673~1.440) | 0.934 |  |  |  |  |  |
| Blood transfusion | |  |  |  |  |  |  | |
|  | No | 1 |  |  |  |  |  |  |
|  | Yes | 1.322(0.828~2.110) | 0.243 |  |  |  |  |  |
| Primary anastomosis | | |  |  |  |  |  | |
|  | No | 1 |  |  | 1 |  |  |  |
|  | Yes | 0.402(0.276~0.587) | **<0.001** |  | 0.543 (0.355~0.831) | **0.005** |  |  |
| Colostomy | |  |  |  |  |  |  | |
|  | No | 1 |  |  |  |  |  |  |
|  | Yes | 0.675(0.445~1.024) | 0.065 |  |  |  |  |  |
| Length of stay (days) | | |  |  |  |  |  | |
|  | ≤17 | 1 |  |  | 1 |  |  |  |
|  | >17 | 1.686(1.112~2.557) | **0.014** |  | 0.961 (0.591~1.561) | 0.872 |  |  |
| Any postoperative complications | | |  |  |  |  |  | |
|  | No | 1 |  |  | 1 |  |  |  |
|  | Yes | 1.758(1.071~2.884) | **0.026** |  | 1.993 (1.140~3.485) | **0.016** |  |  |
| CEA (ng/mL) | |  |  |  |  |  |  | |
|  | ≤11.6 | 1 |  |  | 1 |  |  |  |
|  | >11.6 | 2.685(1.781~4.048) | **<0.001** |  | 1.011 (0.603~1.693) | 0.968 |  |  |
| CA19-9 (kU/L) | |  |  |  |  |  |  | |
|  | ≤53.2 | 1 |  |  | 1 |  |  |  |
|  | >53.2 | 1.996(1.172~3.397) | **0.011** |  | 1.057 (0.590~1.894) | 0.852 |  |  |
| CA125 (U/mL) | |  |  |  |  |  |  | |
|  | ≤15.9 | 1 |  |  | 1 |  |  |  |
|  | >15.9 | 2.226(1.454~3.408) | **<0.001** |  | 1.730 (1.108~2.700) | **0.016** |  |  |
| CA72-4 (U/mL) | |  |  |  |  |  |  | |
|  | ≤9.4 | 1 |  |  | 1 |  |  |  |
|  | >9.4 | 2.685(1.691~4.265) | **<0.001** |  | 2.289 (1.392~3.764) | **0.001** |  |  |
| VFA | |  |  |  |  |  |  | |
|  | Low | 1 |  |  |  |  |  |  |
|  | High | 1.042(0.714~1.521) | 0.831 |  |  |  |  |  |
| IMFA | |  |  |  |  |  |  | |
|  | Low | 1 |  |  |  |  |  |  |
|  | High | 1.327(0.770~2.289) | 0.309 |  |  |  |  |  |
| SMA | |  |  |  |  |  |  | |
|  | Low | 1 |  |  |  |  |  |  |
|  | High | 0.907(0.618~1.329) | 0.616 |  |  |  |  |  |
| SFA | |  |  |  |  |  |  | |
|  | Low | 1 |  |  |  |  |  |  |
|  | High | 0.973(0.663~1.429) | 0.889 |  |  |  |  |  |
| SMD | |  |  |  |  |  |  | |
|  | Low | 1 |  |  | 1 |  |  |  |
|  | High | 0.343(0.215~0.549) | **<0.001** |  | 0.571 (0.329~0.993) | **0.047** |  |  |
| SMI | |  |  |  |  |  |  | |
|  | Low | 1 |  |  | 1 |  |  |  |
|  | High | 0.559 (0.373~0.838) | **0.005** |  | 0.568 (0.359~0.897) | **0.015** |  |  |
| **Abbreviations: BMI, body mass index (weight [kg]/height [m^2^]); LVI, lymphovascular invasion; COPD, chronic obstructive pulmonary disease; CEA, carcino-embryonic antigen; CA19-9; CA125; CA72-4, carbohydrate antigen; VFA, visceral fat area; IMFA, intermuscular fat area; SMA, skeletal muscle area; SFA, subcutaneous fat area; SMD, skeletal muscle density. SMI, skeletal muscle index. Any comorbidities, including cardiovascular disease, cerebrovascular disease, COPD, and diabetes. Any postoperative complications, including obstruction, anastomotic fistula, local infection, thrombosis, cardio-cerebrovascular disease.** | | | | | | |  |  |
|  |  |  |  |  |  |  |  |  |
|  |  |  |  |  |  |  |  |  |
|  |  |  |  |  |  |  |  |  |
| **Bold was used to highlight values that were statistically significant (P<0.05).** | | | | | | |  |  |
